# Recent insights into uptake, toxicity, and molecular targets of microplastics and nanoplastics relevant to human health impacts

**DOI:** 10.1016/j.isci.2023.106061

**Published:** 2023-01-27

**Authors:** Ajmal Khan, Zhenquan Jia

**Affiliations:** 1 Department of Biology, University of North Carolina at Greensboro, 312 Eberhart Building, 321 McIver Street, Greensboro, NC 27412, USA

**Keywords:** Health sciences, Earth sciences, Environmental science, Environmental health, Pollution

## Abstract

Microplastics and nanoplastics (M-NPLs) are ubiquitous environmentally, chemically, or mechanically degraded plastic particles. Humans are exposed to M-NPLs of various sizes and types through inhalation of contaminated air, ingestion of contaminated water and food, and other routes. It is estimated that Americans ingest tens of thousands to millions of M-NPLs particles yearly, depending on socioeconomic status, age, and gender. M-NPLs have spurred interest in toxicology because of their abundance, ubiquitous nature, and ability to penetrate bodily and cellular barriers, producing toxicological effects in cells, tissues, organs, and organ systems. The present review paper highlights: (1) The current knowledge in understanding the detrimental effects of M-NPLs in mouse models and human cell lines, (2) cellular organelle localization of M-NPLs, and the underlying uptake mechanisms focusing on endocytosis, (3) the possible pathways involved in M-NPLs toxicity, particularly reactive oxygen species, nuclear factor-erythroid factor 2-related factor 2 (NRF2), Wnt/β-Catenin, Nuclear Factor Kappa B (NF-kB)-regulated inflammation, apoptosis, and autophagy signaling. We also highlight the potential role of M-NPLs in increasing the incubation time, spread, and transport of the COVID-19 virus. Finally, we discuss the future prospects in this field.

## Graphical abstract

**Figure:**
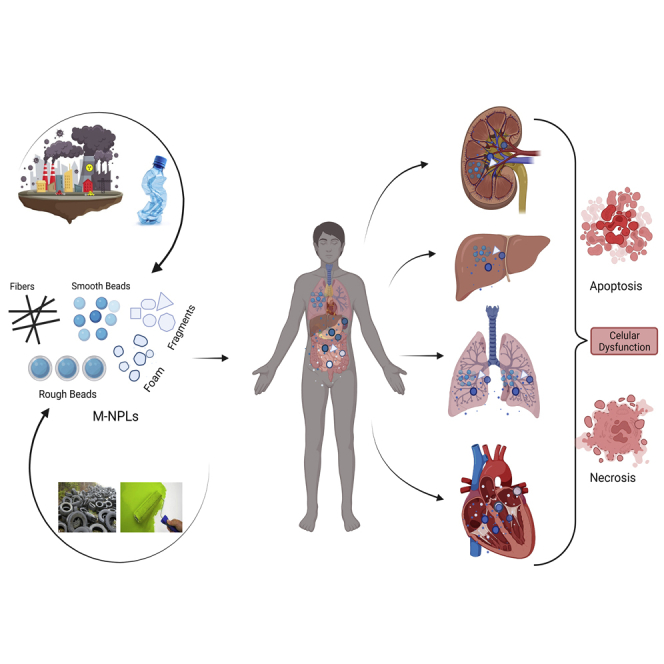

## Introduction

Plastics remain indispensable materials for packaging and products such as pharmaceuticals, cosmetics, textiles, facemasks, and surgical instruments.^1^^,^^2^^,^^3^^,^^4^^,^^5^ The excessive use of plastics is attributed to their versatile properties including high durability, impervious nature, cost-effectiveness and simple manufacturability with low energy demand.^1^^,^^5^ These features render them particularly suitable for manufacturing medical equipment (syringes, intravenous bags, medical equipment packaging, prosthetic joints, artificial limbs, and tissue engineering), food containers and other plastic wares.^6^ However, despite these benefits, plastics have been criticized for being harmful to the environment and human health because of their persistence, ubiquitous nature, and potential to contaminate animal food and drinking sources.^1^^,^^6^ Plastic production grew alongside the industrial revolution and has been increasingly dominant in the consumer sector since its commercialization in the 1930 and 1940s. Global plastic resin production escalated by 620% between 1975 and 2012, amounting to 288 million metric tons (MT) production at that time.^7^ Consequently, plastic waste production rose from 275 million MT in 2010 to 335 million MT in 2017.^8^^,^^9^ Hence, plastics may pose severe threats to human health as their use is mostly not sustainable.^6^ Despite being recyclable, only 8.8% of plastics are recycled in the US.^10^ Plastic waste has an extended half-life and a slow decomposition rate. For instance, single-purpose plastics, such as LDPE bags, have an extended half-life of up to 250 years in a landfill, compost, or soil condition.^11^ By 2025, the ocean area within 50 km of the coasts of 192 countries will accumulate 250 million MT of improperly managed plastic marine garbage.^7^ These long-lasting plastics accumulate and eventually convert into micro and nanoscale-sized plastics (microplastics and nanoplastics) of various structures and chemical forms through physical, chemical, and microbial degradation.^12^^,^^13^^,^^14^

Microplastics and nanoplastics (M-NPLs) are hazardous forms of plastics ubiquitously found in environments ranging from the atmosphere to the hydrosphere. Depending on the structure of the added motifs and primary product, the M-NPLs can exist as fibers, foam, beads, and irregular fragments.^15^ Microplastics with spherical shapes have been reported to produce less harm and inflammatory response in the gut than those with irregular shapes.^16^ Controversial reports exist regarding the size of plastic debris in the environment. However, they are generally classified as; macroplastics (2.5-100 cm), mesoplastics (0.1 - 2.5 cm), microplastics (1000 μm–1 μm), and nanoplastics (<1 μm).^17^^,^^18^^,^^19^ Others have classified microplastics (5000 μm–1 μm) and nanoplastics rather differently (1 μm–1 nm),^20^^,^^21^ or 100 nm–1 nm.^22^^,^^23^ The enormous use of plastic goods has recently been identified as a potential source of M-NPLs pollution, and it has piqued the interest of ecotoxicologists and medical science researchers.^24^ M-NLPs pollution has become a worldwide concern as it poses a major threat to inhabitants of all ecosystems, including human beings. Humans are exposed to M-NPLs by ingesting food and water, inhaling contaminated air, and having dermal contact through cosmetics and pharmaceuticals.^25^ M-NPLs are toxic on *in vivo*, *in vitro*, and environmental exposure in experimental model animals, cells, and various aquatic and terrestrial animal species.^26^^,^^27^^,^^28^

There exists a dearth of knowledge regarding the size, structure, and charge of M-NPLs. The potential toxic effects of M-NPLs on various human organ systems, their mechanism of cellular uptake, and the molecular pathways behind their toxicity have not been discussed adequately, principally because of the dispersed literature available, which also bears many disagreements. This study attempts to discuss in detail: (1) the origin, sources, and potential reservoirs (soil, food, air, water, etc.) of MPLs and NPLs, (2) Potential human exposure routes (inhalation, ingestion, skin or dermal contact) to MPLs and NPLs, (3) accumulation and toxic effects of these M-NPLs in different organ systems, (4) Cellular uptake and toxicity of M-NPLs, (5) the molecular mechanism and pathways of MPLs and NPLs uptake and toxicity, and finally, (6) the future prospects in M-NPLs research.

## Types, sources, and surface alterations of microplastics and nanoplastics

**Figure 1:**
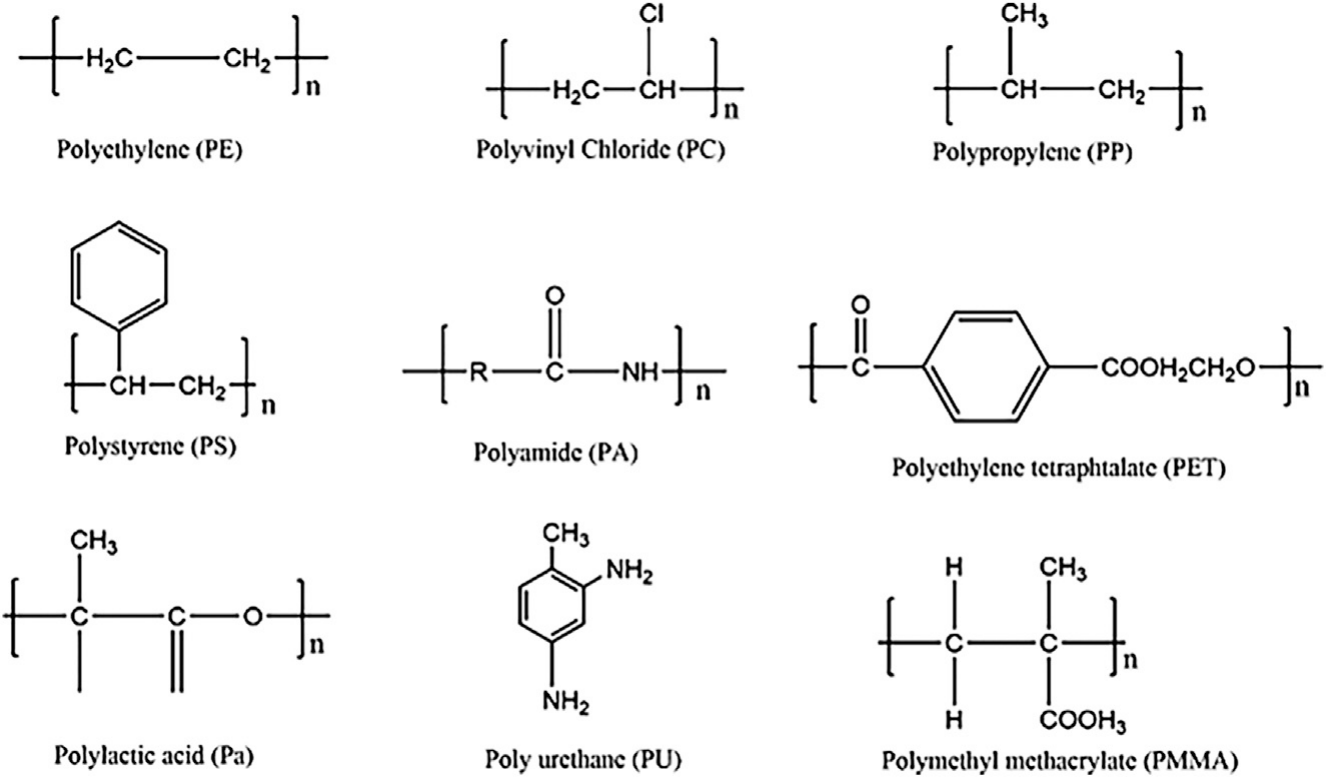
Molecular structure of various M-NPLs found in the environment

Numerous plastic polymers (Figure 1) contribute to M-NPLs pollution, including polystyrene (PS), polylactic acid, polyurethane (PU), polyethylene (PE), polyoxymethylene (POM), polyethylene-terephthalate (PET), polyamides (PA), polymethyl-methacrylate (PMMA), polypropylene (PP), styrene acrylate, polyvinyl chloride (PVC), and styrene acrylate (SA).^29^^,^^30^^,^^31^^,^^32^^,^^33^^,^^34^ The abundance of a specific type of plastic particles in any ecosystem varies with location.^35^^,^^36^^,^^37^

M-NPLs can be derived from numerous sources, such as goods manufacturing, biomedical applications, textiles, industrial pollutants, urban transportation, laundry, and landfills, and are categorized as primary or secondary plastics. Primary plastics are manufactured on an industrial scale to be used as raw materials in consumer polymer goods, either indirectly or directly, whereas their degraded particles deposit as M-NPLs in the environment.^25^ Primary M-NPLs originate from various products, including paints, cosmetics, medical equipment, and packing materials. Secondary M-NPLs form when larger plastic particles degrade into smaller particles via physical (mechanical forces and pressures induced fragmentation), chemical (fragmentation because of photodegradation), and microbial processes^25^^,^^38^ that occur on environmental exposure.^37^^,^^39^^,^^40^ Secondary M-NPLs include fibers from the laundry of synthetic clothing, particles resulting from the abrasion of plastic coatings, and automobile tires.^41^ Based on the size differences, secondary M-NPLs might be considered secondary MPLs or NPLs. Secondary M-NPLs can also be accumulated with inefficient industrial materials processing and faulty waste management systems. For instance, M-NPLs released from disposable polystyrene (PS) coffee cup lids, improper fishing nets, rope disposal, and river run-off contribute significantly to global M-NPLs levels.^40^^,^^42^^,^^43^

M-NPLs modified intentionally or accidentally are more physiologically compatible and have an enhanced ability to interact and traverse biological barriers, producing toxicological effects.^44^ These alterations include adding or exposing an amine or carboxyl group to M-NPLs, making the shape more biologically compatible and the color more palatable, or resulting in M-NPLs with a net positive or negative charge. Birds consume plastics by mistaking them for food^45^^,^^46^due to their color and smell, often falling into the DMS (dimethyl sulfide) smell trap produced by the digestion of algae (mostly attached to plastics) by krill (a common prey for birds).^47^ Furthermore, M-NPLs containing additional moieties bind to external and internal body proteins, such as albumin, fibrinogen, and globulins, as reported for M-NPLs collected from circulation. This interaction forms a complex structure known as the corona, which protects the integrity of M-NPLs within and outside organisms' bodies. This, in turn, extends the half-life and blood circulation time of M-NPLs,^48^^,^^49^ resulting in increased cellular uptake and toxicity.^50^ Positively charged PS-NH_2_ have been reported to possess the highest toxicity to biofilms^51^ because they exhibit a high propensity for attaching to algae than NPLs with negative charges.^52^

## Routes of HUMAN exposure to microplastics and nanoplastics

Living organisms, especially humans, are exposed to M-NPLs through three main routes: ingestion, inhalation, and dermal contact.^25^ Hence, M-NPLs can enter the body by ingesting contaminated food and water, inhaling contaminated indoor and outdoor air, and cutaneous exposure to M-NPLs through dust, clothing, and personal care items.^53^ One less discussed aspect of exposure to M-NPLs is the entanglement of specific marine species in plastic debris, which causes physical and biological injuries.^54^ In 1997, over 200 aquatic animal species were estimated to suffer from entanglement in plastic debris.^55^ However, the worst scenario can be expected in the coming years, as plastic debris accumulation in marine ecosystems continues to rise exponentially. Moreover, exposure to M-NPLs from medical treatments and equipment is one of the least researched areas. For example, plastics used in surgical equipment, rectal and vaginal suppositories, implantable cardioverter-defibrillator (ICD), hip replacement implants containing various forms of ethylene {ultra-high molecular weight polyethylene (UHMWPE), cross-linked polyethylene (XLPE), conventional polyethylene (CPE), or high-density polyethylene (HDPE)}, breast implants containing polyurethane foam, repairing damaged bone and cartilage by BioSphere need to be investigated for their release of M-NPLs into the body.^56^ Because all the medical procedures mentioned above involve compromised subjects, with most of the physical barriers to M-NPLs entry already bypassed, they can serve as potent ways to M-NPLs exposure. More importantly, short invasive medical-based exposure to M-NPLs may result in high accumulation and deteriorating effects compared to environmental exposure.^56^ To date, M-NPLs have been detected in both natural and bottled waters,^57^^,^^58^ air,^59^ soil,^60^ sediments,^21^ and animal tissues (humans included)^61^ (Table 2), indicating their possible transfer through the food chain.^62^ Further details about the routes of M-NPLs exposure can be found in a scoping review by Rahman et al.^63^

### Ingestion of microplastics and nanoplastics

M-NPLs are an emerging hazard to food security, water security, and human health.^64^ The primary route to M-NPLs exposure in all animals, particularly humans, is ingesting contaminated food and water.^65^^,^^66^ Aquatic and terrestrial animals, such as birds, ingest a huge load M-NPLs accidentally or by confusing plastics for food. Nearly every seabird may be consuming plastics by 2050.^67^ Pacific oyster larvae are reported to readily ingest NPLs.^66^ Moreover, ingestion of NPLs by *Artemia franciscana* (brine shrimp) has been shown to be independent of the presence or absence of food,^68^ leading to NPLs adsorption and bioaccumulation in the mandible, stomach, gut, tail, gut, and appendages.^68^ Human beings consume M-NPLs present in take-out food and their containers. M-NPLs ingestion from food containers could be as high as 203 pieces per person per week.^69^ Food contaminated with M-NPLs includes sugar, salt, bottled water, and almost all seafood, such as bivalves, oysters, fishes, and crustaceans.^57^^,^^66^^,^^70^^,^^71^^,^^72^^,^^73^^,^^74^^,^^75^ There have also been reports of unintentional human NPLs ingestion from sources such as food, drinks, and water.^76^ These plastic particles accumulate in tissue of various organisms and are transferred to human bodies as soon as they are consumed as food.^77^ The worst-case scenario is that MPLs are fragmented into NPLs, as seen in Antarctic krill, making absorption and bioaccumulation more probable.^78^ Even though food and water quality is closely monitored, it is estimated that every US resident consumes 39,000–52,000 M-NPLs particles per year.^65^ An even worse scenario can be expected for people living in underdeveloped countries. It is also worth mentioning that the dust that settles on food containers, packaging, and serving plates could be a more significant source of MPLs than the actual food. This M-NPLs dust can also contaminate food while opening plastic food packaging.^79^^,^^80^ Because ingestion of M-NPLs in food and water is one of the primary route exposures, the effect of cooking and temperature on the M-NPLs in food and water has been investigated, showing that cooking resulted in lower MPLs levels (−14%) in cooked tissues compared to raw ones. Also, the MPLs recorded in cooking water were smaller than in raw mussels, implying that proper cooking might degrade M-NPLs and may alter M-NPLs induced toxic effects.^81^ Various mechanisms by which M-NPLs enter, and cross mucosa and the GIT system have been described later in this review.

### Inhalation of microplastics and nanoplastics

Inhalation is also one of the significant pathways of M-NPLs entry into the body.^81^ Several studies have found fibrous MPLs in the atmosphere. These M-NPLs end up in the air we breathe after being released from synthetic clothing and textiles, building materials, plastics, waste incineration, and landfilling.^2^^,^^53^^,^^82^ Several studies have found M-NPLs in human samples, including lungs and sputum.^61^^,^^83^^,^^84^ Jenner et al.^61^ detected 39 M-NPLs particles (size ≥3 μm) in 11/13 human lung tissues with an average of 1.42 ± 1.50 MPLs/g of tissue. Amato-Lourenco et al.^83^ also studied 20 pulmonary tissue samples from the left lung of non-smokers and detected 33 polymeric and 4 fibers in 13/20 samples with an average size of 8.12–16.8 μm in a mean weight of 3.28 g of tissue. Furthermore, Huang et al.^84^ found 18.75 to 91.75 particles/10 mL of sputum samples from 22 patients suffering from respiratory diseases. Depending on the characteristics of the particles and the residents’ lifestyle, these M-NPLs could have a variety of fates after inhalation, including systemic circulation and transport to various tissues, cellular internalization, and removal from the body.^85^^,^^86^

Besides the potentially toxic effects of M-NPLs, they also serve as carriers^53^^,^^63^^,^^87^ of other contaminants. NPLs have also been shown to carry chemical and biological contaminants owing to their low polarity and high surface roughness.^88^ After entry into air passageways, these plastics and their loaded toxicants can get easily absorbed into the fine alveolar epithelium and produce local inflammation. They are then translocated to the systemic circulation, creating systemic problems or stimulating pro-inflammatory factors' production, producing systemic inflammation.^53^^,^^63^^,^^89^^,^^90^^,^^91^ This inflammation is touted as dust overload.^53^^,^^63^ Loaded chemical and biological contaminants can also have synergistic local and systemic effects, resulting in serious illnesses such as cytotoxic and genotoxic effects, asthma-like reactions, granulomatous modifications in bronchial tissues, persistent pneumonia, and extrinsic allergic alveolitis.^53^^,^^63^

### Dermal exposure to microplastics and nanoplastics

Humans and other organisms also absorb M-NPLs through dermal contact with topical agents such as cosmetics, body wash, topical pharmaceuticals, surgical and prosthetic devices, and incidental indoor or outdoor occupational exposure. MPLs have been found in hand and face washes,^3^^,^^92^ facemasks,^92^ sunscreens,^4^ and toothpaste^93^ in the form of beads that are absorbed and cause skin injury.^94^ M-NPLs have also been excessively used in prosthetic equipment, surgical instruments, and other pharmaceutical agents.^4^^,^^95^ M-NPLs have not been found to cross the subcutaneous barrier under standard conditions; however, they have been shown to accumulate in hair follicles, and PS-NPLs have been reported to be taken up by Langerhans cells.^96^^,^^97^^,^^98^ In addition, skin that has been damaged because of an injury or illness is more porous compared to normal skin and may serve as a route for unintentional M-NPL entry.^99^

## Organ accumulation and cellular uptake of microplastics and nanoplastics

Following exposure through ingestion, inhalation, or dermal routes, M-NPLs can be taken up by various cells (Table 1) and accumulate intracellularly and in multiple tissues/organs.^25^ Intracellular accumulation occurs when these pollutants interact with cell membrane components (receptors, lipids), resulting in bioaccumulation.^121^ All bodily systems have shown traces of these particles in them.

**Table 1:** Localization of M-NPLs

M-NPLs (Size)	StudyType/cells/species	Results	Reference
PS-NPLs (100 & 500 nm)	*In vitro*, Human Umbilical Vein Epithelial cells (HUVECs)	• Both sizes interacted with HUVCs • Only 100 nm were internalized and initiated autophagy.	Lu, et al., 2022^100^
PS-NPLs, PS-COOH, PS-NH_2_ (100 nm)	Both *in vitro* and *in vivo*, Human intestinal epithelial cells (Caco-2) and Specific pathogen-free (SPF) BALB/c mice (Male, 6 weeks)	• NPLs accumulated in mice tissues (liver, spleen, lung, kidney, small intestine, large intestine, testis, and brain) • Macropinocytosis and clathrin-mediated endocytosis were the main routes for mediating the uptake of NPLs in intestinal cells. • PS-NH_2_ and PS-COOH were observed to be more suitable for entering cells	Xu et al., 2021^101^
PS M-NPLs (0.5 μm)	Both *in vitro* and *in vivo*, Granulosa cells and Female Wistar rats, 6 weeks old, weighing ∼180 g	• PS-NPLs were internalized by Granulosa cells	An et al., 2021^102^
Polyethylene terephthalate (PET) NPLs (200 nm)	*In vivo*, BALB/c female mice	• The digestive tract and gills were the primary sites of accumulation • NPLs distributed to the Liver, Spleen, lung, heart, and blood vessels	Gao et al., 2022^103^
Polystyrene Microsphere (70 nm, 5 μm, and 20 μm)	*In vivo*, Zebrafish (*Danio rerio*)	• 5 μm particles accumulated in various tissues (gills, gut, and liver) • Variations in liver metabolomics	Lu et al., 2016^104^
Green, fluorescent PS-MPLs (0.1 and 1 μm)	Both *In vitro* and *in vivo*, Human Liver cell lines (HL7702) and SPF male C57 mice	• 0.1 μm (not 1 μm) PS-MPLs entered liver cells and accumulated in the liver	Shen et al., 2022^105^
PS-NPLs (20 nm)	*In vivo*, Zebrafish embryo (*Danio rerio*)	• PS-NPLs accumulated in brain tissue	Sökmen et al., 2020^106^
PS-MPLs (3.54 ± 0.39 μm)	*In vitro* Human embryonic kidney 293 (HEK293)	• PS-MPLs adhered to cells and were internalized	Chen et al., 2022^107^
PS-MPLs (213.7 ± 8.2 nm)	*In vitro*, Human gastric epithelial (GES-1) cells	• PS-MPLs interacted through halogen bonds with cell membrane • PS-MPLs also interacted with cellular proteins	Qin et al., 2022^108^
PS-*M*-NPLs (50 nm, 500 nm, and 5 μm)	*In vitro*, model cell membranes and rat basophilic leukemia (RBL-2H3) cells	• PS M-NPLs (50 nm) were internalized by clathrin- and caveolae-mediated endocytosis • PS M-NPLs (500 nm) were internalized by macropinocytosis • These particles also interacted with lysosomes and were released via lysosomal-mediated exocytosis	Liu et al., 2021^109^
Rhodamine-labeled polystyrene beads (20 nm)	*In vivo*, Time-pregnant Sprague-Dawley rats	• Particles were observed in various tissues (placenta, fetal liver, lungs, heart, kidney, brain, and spleen)	Fournier et al., 2020^110^
PS M-NPLs (50 nm, 100 nm and 1 μm)	Both *In vitro* and *in vivo*, Hemocytes and *Mytilus galloprovincialis*	• M-NPLs size dependently accumulated in *M. galloprovincialis* • PS-NPLs (50 nm) significantly translocated to hemolymph • Hemocytes internalized PS M-NPLs through different endocytosis pathways	Sendra et al., 2020^111^
PS M-NPLs ((0.5μm, 4μm, 10μm)	Both *in vitro* and *in vivo*, Germ cells (GC), Leydig cells (LC), and Sertoli cells (SC) and Male BALB/C mice	• PS M-NPLs (4 and 10 μm) accumulated in testis	Jin et al., 2021^112^
PS-NPLs (43.67 ± 1.08 nm)	Bovine oviductal epithelial cells (BOEC) and Human colon fibroblasts (HCF)	• NPLs internalized through ATP-independent pathway • NPLs were also released rapidly in the culture medium • NPLs traverse the cells by passive translocation	Fiorentino et al., 2015^113^
High-density polyethylene (HDPE) particles (0–80 μm)	*In vivo*, blue mussel (*Mytilus edulis* L.)	• HDPE particles were taken up by gills and stomach and were transported to digestive glands	von Moos et al., 2012^114^
PS-NPLs (100, 200, 500, 1000nm) & Negative charged PS-NPLs (100, 500, 1000 nm)	*In vitro*, Human induced pluripotent stem cells (hiPSCs)	• hiPSCs internalized PS-NPLs	Jeong et al., 2022^115^
PS-NPLs (50 nm)	*In vitro*, Human intestinal epithelial cells (Caco-2)	• PS-NPLs accumulated in the cell and nucleus	Domenech et al., 2021^116^
PS-MPLs (1, 2, 3, 4 and 5 μm)	*In vivo*, *P. helgolandica var. tsingtaoensis* and *S. quadricauda*	• PS-MPLs (1-2 μm) were taken up by cells • PS-MPLs (3-5 μm) were not engulfed by cells	Chen et al., 2020^117^
PS M-NPLs (460 nm, 1, 3, 10, 40, & 100 μm)	*In vitro*, Human Dermal Fibroblasts (HDFs), Human Peripheral Blood Mononuclear Cells (PBMCs), Red blood cells (RBCs) & the Human Mast Cell line (HMC-1)	• The uptake of PS-particles occurred through endocytosis and phagocytosis • van der Waals forces enhanced attachment of PS-particles to RBCs • PS-particles were observed after 24 h in the cytoplasm of HDFs and PBMCs • PS-FITC particles were engulfed by neutrophils and macrophages but not lymphocytes	Hwang et al., 2020^118^
Low-density polyethylene (LDPE)-MPLs (50–500 μm)	*In vivo*, Catfish (*Clarias gariepinus*)	• PS- MPLs were accumulated in the GIT tract	Tongo et al., 2022^119^
PS-NPLs (44 and 100 nm)	*In vitro*, Gastric adenocarcinoma (AGS) cells	• 44 nm PS-NPLs accumulated rapidly as compared to 100 nm in AGS cells • Both particles underwent clathrin-mediated endocytosis and an energy-dependent internalization.	Forte et al., 2016^120^

### Accumulation of microplastics and nanoplastics in organs

M-NPLs build up in the bloodstream^122^ and are distributed to various organs. For instance, plastic particles (700 nm) have been detected and measured in blood samples of 17/22 healthy human blood donors using gas chromatography/mass spectrometry. The most common were polyethylene terephthalate, polyethylene, polymers of styrene, and poly (methyl acrylate), representing a 1.6 μg/mL mean of the total quantifiable concentration M-NPLs in the blood.^122^ A study involving exposure of zebrafish to NPLs labeled with near-infrared (NIR) surface-enhanced Raman scattering (SERS) reported NPLs entry into the bloodstream through the dermal route, and SERS signals were later detected in zebrafish’s hearts and blood vessels. The digestive tract and gills were the primary sites of accumulation.^123^ Xu et al.^101^ used PS-NPLs (unmodified green fluorescent) and two different modified nanoplastics, PS-COOH (carboxyl-modified) and PS-NH_2_ amino-modified (10 mg/mL, 100 nm), to investigate the systematic toxicity and molecular mechanism of NPLs internalization in mice (BALB/c). Biofluorescence imaging revealed that NPLs accumulated preferentially in the testis, stomach, kidney, and small and large intestines. PS-NH_2_ and PS-COOH were found in the lungs, with PS-COOH also found in the brain. Confocal microscopy also revealed the presence of NPLs in testicular tissue, colon, kidney, spleen, and lung.^101^ Atamanalp et al.^124^ provided data for tissue based MPLs assessment in fishes, which investigated the presence, composition, and characterization of MPLs in commercial fish species, red mullet (*Mullus barbatus*) and Pontic shad (*Alosa immaculata*). MPLs were isolated from tissues utilizing the flotation method, then counted and classified by form, size, and color. The abundance of MPLs in fish tissues was determined using ATR–FTIR spectroscopy which amounted to 40% in the gastrointestinal tract, 30% in the gills, and 7% in the brain. Regardless of fish species, MPLs were primarily fibrous (51%), black (49%), and 50–200 μm in size (55%). Polychloroprene (18.8%) and polyamide (15%) were found to be the most common among the nine polymers identified.^124^ In another study, Zebrafish (*Danio rerio*) exposed to PS-MPLs for 7 days resulted in liver uptake and accumulation of both 5 μm and 70 nm particles.^104^ PS-MPLs (0.1 μm) also accumulate in the liver after being taken up from the bloodstream.^105^ After the microinjection of M-NPLs into the zebrafish embryo (*D. rerio*), a transmission electron microscope (TEM) revealed the presence of 20 nm PS-NPs in the brain tissues.^106^ M-NPLs have also been reported in the kidney of 11 commercial fishes.^125^ Furthermore, 200 nm nanoplastics were detected in the lungs, spleen, kidney, and heart after being intravenously injected.^103^ Several studies have also reported the presence of M-NPLs in human samples such as human blood, lung, sputum, saliva, placenta, and feces, as summarized in Table 2. All these studies show that M-NPLs have the potential to accumulate in almost all the body’s organs on entry into the body.^123^

**Table 2:** Presence of M-NPLs particles in human samples

Human Tissue/sample	Sample Collection	Type of M-NPLs Detected	Size	Quantity	Detection Method	Filter size	Reference
Blood	Whole blood was obtained by venipuncture from 22 anonymized, healthy, non-fasting adult volunteers	PMMA (methyl methacrylate), PP (2,4-dimethyl-1-heptene), PS (styrene), PS (styrene trimer), PE (1-decene), PE (1-undecene), PET (dimethyl terephthalate)	≥700 nm	1.6 μg/mL (Mean quantifiable concentration)	Double shot pyrolysis - gas chromatography/mass spectrometry (Py-GC/MS)	Filtered through a glass fiber filter with a 25 mm diameter and a 700 nm mesh size.	Leslie et al., 2022^122^
Lungs	13 lung tissue samples	Polyacrylonitril (PAN), polyethylene (PE), polyester (PES), polyethylene terephthalate (PET), polymethylmethacrylate (PMMA), polypropylene (PP); PS, polystyrene (PS), Polytetrafluoroethylene (PTFE), polyurethane (PUR), styrene-ethylene-butylene co-polymer (SEBS), thermoplastic elastomer (TPE)	≥3 μm	39 MPLs were identified within 11 of the 13 lung tissue samples with an average of 1.42 ± 1.50 MP/g of tissue	μFTIR spectroscopy	The mercury cadmium telluride (MCT) detector, which was cooled, enabled precise particle analysis up to a 3 μm size range.	Jenner et al., 2022^61^
Lungs	20 Pulmonary tissue samples from a left lung non-smokers dead individual (mostly due to respiratory system problems)	Polypropylene, Cotton, Polyethylene, Cellulose acetate, Polyvinyl chloride, Polyethylene-co-polypropylene, Polystyrene, Polystyrene-co-polyvinyl chloride, Polyamide	Polymeric (<5.5 μm), Fibers (8.12-16.8 μm)	Polymeric particles (n = 33) & fibers (n = 4) were observed in 13/20 in a mean weight of 3.28 g tissue samples.	Raman spectroscopy	25 mm diameter & 0.45 μm pore size silver membrane filter	Amato-Lourenço et al., 2021^83^
Sputum	Sputum samples from 22 patients suffering from respiratory diseases	21 types: Acrylates, acrylonitrile butadiene, alkyd varnish, ethylene vinyl acetate, polyacetal, polybutadiene, polyester, polyethylene Chlorinated polyethylene, polyimide, chlorinated polyisoprene, polymethylmethacrylate, polypropylene, polysulfones, polyurethane, polyvinyl alcohol, polyvinylchloride, rubber, silicone, polycarbonate, polytetrafluoroethylene	20−500 μm	The median (interquartile range, IQR) level of the total number of microplastics was 39.5 particles/10 mL (18.75–91.75 particles/10 mL)	optical microscope (HDS200G stereoscopic microscope, Agilent 8700 laser infrared imaging spectrometer, and Fouriertransform infrared microscope	0.45 μm pore silver membrane and particles larger than 100 μm on the filter were screened.	Huang et al., 2022^84^
Saliva, Head Hairs, Face skin, Hand skin	8000 samples from 2000 participants	Polyethylene, polyethylene terephthalate, polypropylene, polystyrene, polyvinyl chloride,	(Length (L) ≤ 100 μm; 100 < L ≤ 250 μm; 250 < L ≤ 500 μm; L > 500 μm)	16,000 particles/8000 samples, (>7000, or, on average, >3.5 MPLs per individual per day)	Binocular Microscopy and micro-Raman spectroscopy	2 μm Blue band filters (s&S)	Abbasi, et al., 2021^126^
Placenta	6 human placentas from women with physiological pregnancies	Polypropylene (Particles 2, 10, and 11), Paint/coating/dye MPLs (Particles 1, 3–9, and 12)	5–10 μm	12 fragments in a 23-gram sample out of the total ∼600 g of Placenta	Optical Microscopy for detection and morphological characterization of MPLs, Raman Micro-spectroscopy analysis for identification	Whatman GF/A Filter membrane with 1.6 μm pore size	Ragusa et al., 2021^127^
Stool	Stool samples from 8 individuals	PA = polyamide; PC = polycarbonate; PE = polyethylene; PET = polyethylene terephthalate; POM = polyoxymethylene; PP = polypropylene; PS = polystyrene; PU = polyurethane; PVC = polyvinyl chloride	50–500 μm	median microplastic concentration was 20 pieces (IQR, 18 to 172 pieces) per 10 g of stool.	Fouriertransform infrared (FT-IR) micro-spectroscopy	50-μm metal sieve,1 sample per individual, smaller or larger than 50-500 μm were not detected	Schwabl et al., 2019^128^

### Transport of microplastics and nanoplastics inside the body

The transport of M-NPLs through the body’s vascular system, paracellular and transcellular pathways can enhance the distribution of M-NPLs in the body.^25^^,^^129^^,^^130^^,^^131^ It is reported that NPLs, due to their smaller sizes, are best suited to penetrate biological membranes and exert more toxicological effects than MPLs.^21^^,^^101^^,^^132^ Moreover, differences exist in the rate of the cellular internalization of NPLs, with smaller particles (50 nm) internalizing faster than larger particles (1 μm).^111^ Oral exposure to NPLs causes nanoplastics to accumulate in the luminal cavities of the digestive system. Depending on their adherence to the gastrointestinal mucus membrane, MPLs can be engulfed by specialized M-cells of the intestinal lymphoid tissue (Peyer’s patches) or directly absorbed into the gastrointestinal mucosa.^133^ NPLs can easily pass the intestinal mucosa of rats by transcellular and paracellular transport.^134^ Translocation of NPLs from maternal to fetal tissues through the placenta suggests the importance of M-NPLs translocation in their toxicity.^110^ It has also been shown that various sizes of NPLs can be translocated into the hemolymph.

M-NPLs have been reported to disrupt tight junctions, promoting paracellular translocation.^111^^,^^135^ Xu et al.^101^ observed that occludin, a plasma membrane protein present in tight junctions,^136^ and zonula occludens-1 (ZO-1), another tight junction protein, were reduced after 28 days of oral exposure to NPLs, suggesting the disruptive role of these NPLs in tight junctions.^137^ Occludin and ZO-1 function together as an integral part of tight junctions.^138^ PS-MPLs (300 ng/mL) have been shown to deplete the zonula occludens-2 proteins and α1-antitrypsin in human embryonic kidney 293 (HEK293) cells, causing impaired kidney barrier integrity and an increased likelihood of developing acute kidney injury.^107^ Further support for this disruptive role of NPLs was aided by the upregulation of Matrix metallopeptidase 9 (MMP-9) protein after oral exposure to NPLs [117]. MMP-9 could degrade tight junctions and extracellular matrix.^139^^,^^140^ MPLs have also been reported to disrupt intestinal barriers by employing the same mechanism.^112^^,^^113^ The endocytosis and lysosome-mediated exocytosis of M-NPLs are discussed below, and these mechanisms might also be involved in the transcellular transport of M-NPLs.

### Interaction of microplastics and nanoplastics with cell membranes

M-NPLs have been observed to interact and adhere with the cell membrane, as observed for PS-MPLs through light and fluorescent microscope images of human embryonic kidney 293 (HEK293) cells treated with PS-MPLs (3-300 ng/mL).^107^ Qin et al.^108^ used molecular dynamics (MD) simulations to investigate how these M-NPLs adhered to the cellular bilipid membrane and reported that PS-MPLs, both pristine and chlorinated, interact with the lipid membrane’s hydrophilic barrier and quickly insert themselves into the membrane’s hydrophobic tail region. PS-MPLs utilize hydrogen bonds to interact with the lipid bilayer. In contrast, chlorinated PS-MPLs use halogen bonds to interact with the cell membrane^108^^,^^141^ and improve their cellular internalization.^142^ The formation of C-Cl bonds (halogen bonds) might also be responsible for the enhanced cell membrane permeability.^108^ Liu et al.^109^ further describe the interaction of M-NPLs (PS50, PS500, and PS5000 nm (PS-MNPLs)) with the cellular membrane by using rat basophilic leukemia (RBL-2H3) cells and model cell membrane.^143^ Their results indicate that hydrophobic and van der Waals interactions, with electrostatic forces, play a role in PS particle cell membrane interactions.^109^ These findings suggest that M-NPLs interact with the cell membrane via various bonds and molecules, and this interaction aids in M-NPLs internalization.

### Uptake of microplastics and nanoplastics by cell organelles

As shown in Table 1, M-NPLs can be taken up by various cells, including human umbilical vein epithelial cells (HUVECs),^144^ human intestinal epithelial (caco-2),^101^ rat basophilic leukemia (RBL-2H3) cells,^109^ bovine oviductal epithelial cells (BOEC) and Human colon fibroblasts (HCF).^113^ Following internalization, M-NPLs can interact with cellular organelles such as the nucleus,^116^ lysosomes,^109^ mitochondria,^145^ ribosomes,^146^ and others.

### Lysosomes

Various studies have reported the interactions of M-NPLs with lysosomes and their toxic effects. After short-term exposure of Caco-2 cells to PS-NPLs and y-PS-NPLs, TEM microscopy demonstrated an increase in electron-dense vacuoles and lysosomes at a concentration of 6.5 μg/cm^2^. z stack imaging of RBL-2H3 cells treated with PS-NPLs 50, PS-MPLs 500, and PS-MPLs 5000 nm for 6 h showed that PS-NPLs 50 and PS-MPLs 500 were mainly distributed in lysosomes following internalization.^109^ An et al.^102^ studied a 90-day effect of PS-MPLs (0.5 μm) in thirty-two female Wistar rats treated with various concentrations (0, 0.015, 0.15, and 1.5 mg/d) and detected PS-MPLs, via TEM, on the surface of the lysosomes for rats treated with a 0.15 mg/d dose.^102^ Additives-free high-density polyethylene (HDPE) MPLs ranging in size from 0-80 μm have been found in the lysosomal system of the blue mussel *Mytilus edulis L.* after 3 h of exposure (in a 96-h exposure study). Following fusion with lysosomes, numerous particle aggregates were detected in the lumina of primary and secondary ducts and the digestive gland tubules, indicating lysosomal features. In addition, after 6 h of MPLs treatment, there was a significant rise in granulocytoma development and a decrease in lysosomal membrane stability (LMS). Eosinophilic granulocyte vacuoles harboring MPLs were discovered in the connective tissue of digestive glands, ducts, and tubules of these granulocytomas. Moreover, the disruption of the lysosomal membrane increased significantly after 96 h. Hence, these findings point to a highly distinct pattern of consecutive MPLs’ effects on digestive epithelial cells that is time-dependent (particle absorption after 3 h, granulocytoma formation after 6 h, and lysosomal disruption after 96 h).^114^ Similarly, MPLs have been predominately found in vesicles, including lysosomes, in GES-1 cells treated with chlorinated PS-MPLs and non-chlorinated PS-MPLs.^108^ According to a recent study, an excess of NPLs in the lysosomes can result in osmotic flow and lysosomal expansion, resulting in cell death.^115^ Furthermore, lysosomal associated membrane protein (LAMP-2) and lysosomal hydrolase protein (CTSB) were also decreased in 100 nm PS-NPLs exposed HUVECs, suggesting that the 100 nm PS-NPLs resulted in lysosomal dysfunction, which could explain the impairment of autophagic flux and failure in degradation of 100 nm PS-NPLs.^144^ These studies suggest that M-NPLs can accumulate and affect lysosomal-associated cellular activities following cellular internalization.

### Mitochondria

Environmental contaminants, including M-NPLs, have been observed interacting with mitochondria, hence faulting mitochondrial function.^147^ Several studies have found the subcellular localization of NPLs in mitochondria and their effects on mitochondrial function.^148^ However, microplastics have not yet been reported to accumulate in mitochondria, but their interaction and subsequent adverse biological effects have been observed.^145^ TEM images of GES-1 cells show mitochondrial cristae’s disappearance after exposure to 100 μm chlorinated MPLs.^108^ Moreover, 24 h of exposure to 50 nm PS-NPLs and y-PSNPLs resulted in mitochondrial cristae swelling at 6.5 μg/cm^2^ concentration.^116^ In a study by Jeong et al.*,*^145^ the size-dependent effects of polystyrene microbeads were investigated in *Brachionus koreanus* (*Monogonont rotifer*) and JC-1 (5,5′,6,6′-tetrachloro-1,1′,3,3′-tetraethylbenzimi- dazolylcarbocyanine iodide) was used to examine the impact of these beads on mitochondria. JC-1, a lipophilic cation that may infiltrate the mitochondrial membrane, is a diagnostic marker of mitochondrial membrane integrity. Intact mitochondrial membranes with increased mitochondrial membrane potential (MMP) accumulate JC-1 and generate red fluorescence, whereas MMP loss is recognized by the accumulation of JC-1 monomers, which emit green fluorescence.^149^ The above-mentioned study used 200 rotifers treated with 0.05, 0.5, and 6 μm polystyrene microbeads (10 μg/mL) for 24 h and then exposed to 5 μM of JC-1 for 30 min before being evaluated using fluorescence microscopy and spectrophotometry. A decrease in the red/green ratio at 0.05 and 0.5 μm exposed group versus the control and 6 μm group indicated a size-dependent decrease in mitochondrial membrane potential.^145^ Similarly, PS-NPLs can affect the mitochondrial membrane potential, basic respiratory capacity, and ATP generation of spleen leukocytes.^150^ Chlorinated PS-MPLs have also been reported to induce mitochondrial dysfunction hence affecting mitochondrial membrane potential, demonstrated by the accumulation of JC-1 monomer.^108^ These findings conclude that M-NPLs target mitochondria, affecting all biochemical energy pathways of the cell.

### Nucleus

M-NPLs are also reported to affect the nucleus. In a recent study, Caco-2 cells were exposed to fluorescent y-PS-NPLs and non-fluorescent PS-NPLs with diameters of 50 nm for 2 h (1.5 × 10^5^ cells planted in 12-well plates and exposed to 0 μg/cm^2^, 0.26 μg/cm^2^, and 6.5 μg/cm^2^PS-NPLs or y-PS-NPLs).^116^ After 24 h of exposure, confocal microscopy revealed the presence of these plastic particles inside the cells treated at all concentrations. TEM results showed that y-PSNPs, in particular, were present in the cell nucleus at all doses. The control group had normal cell morphology, including a well-organized nucleus, nucleolus, and cellular membranes. However, the treated group presented dark-colored electron-dense structures in the perinuclear area, which indicates that PS-NPLs treatment induced structural changes in the nucleus.^116^ M-NPLs are also reported to damage DNA inside the nucleus.^25^ For example, exposure to low-density polyethylene (LDPE) microplastics (11-13 m) was able to induce genotoxicity with single and double-strand DNA breaks in the peppery furrow shell clam, *Scrobicularia plana*.^151^

### Ribosome

M-NPLs have been shown to influence the abundance of ribosomal protection protein genes, tetM, tetO, and tetQ, linked to the risk of antibiotic resistance gene propagation in biological phosphorus removal systems.^152^ In a study by Zhang et al.*,*^146^ *Microcystis aeruginosa* was subjected to physiological analysis and whole-transcriptome sequencing after being exposed to the antagonism of NPLs (50–100 nm) and multi-walled carbon nanotubes (MWCNTs, diameters of about 20–40 nm and lengths of 40–60 μm) at various concentrations (5 + 5, 10 + 10, 20 + 20, 50 + 50 mg/L). When compared to the control group, tRNA genes were significantly downregulated. T1 (less than 50 mg/L Nano-PS) had a significant increase in ribosomal protein genes, which could explain the upregulation of structural components like the ribosome and ribonucleic-protein complex, with functional consequences such as rRNA binding and translation. Surprisingly, a reduction in total protein concentration was observed. This could be attributed to the selective overexpression of ribosomes to provide a foundation for cell proliferation and metabolism to withstand external stress. Furthermore, for T2 (50 mg/L MWCNTs), the downregulation of tRNA-related genes could have directly impacted anion binding and subsequent metabolic processes.^103^ Hence, these reports indicate that M-NPLs affect ribosomes and the proteins involved in their synthesis.

### Endoplasmic reticulum

Qu et al.^153^ observed that PS-NPLs (1 μg/L) increase unfolded proteins in the endoplasmic reticulum (ER) by triggering p38 MAPK signaling in *Caenorhabditis elegans.* PS-NPLs particles increase the expression of PMK-1, which encodes for p38 MAPK. PMK-1 in the intestine regulates the response to PS-NPLs by acting upstream to two transcriptional factors (ATF-7 and SKN-1), which act upstream to XBP-1, a vital regulator of the ER unfolded protein response (ER-UPR). In nematodes exposed to NPLs, PMK-1, ATF-7, SKN-1, and XBP-1 are reported to cause the induction of intestinal ER-UPR. As a result, activating XBP-1-mediated ER-UPR and intestinal p38 MAPK signaling may mediate a protective response to NPLs.^153^ Furthermore, autophagic and ER stress-related metabolic changes were observed in bronchus epithelial (BEAS-2B) cells exposed to PS-NPLs. Such changes attribute to the regulation of cell resistance to cytotoxic effects. These modifications include increasing the concentration of amino acids and tricarboxylic acid cycle (TCA) intermediate metabolites.^154^ All these studies suggest that M-NPLs have a significant impact on ER.

### Mechanisms of internalization of microplastics and nanoplastics

#### Endocytosis

Even though M-NPLs have been observed to enter the cell by disrupting cell membrane integrity, there are still reports that M-NPLs utilize various molecular pathways to get internalized without affecting cell membrane integrity, such as internalization by intestinal mucosa.^155^ They may interact with membrane transporters or directly cross the cell membranes. Cells may use a variety of ways to internalize particles that are too large or polarized to pass through the membrane. This is evidenced by M-NPLs inside cells with intact cell membranes, implying that M-NPLs utilize transport mechanisms for internalization.^101^ Non-phagocytic cells usually employ clathrin and caveolae-mediated endocytosis, clathrin and caveolae-independent endocytosis, and macropinocytosis.^109^

**Figure 2:**
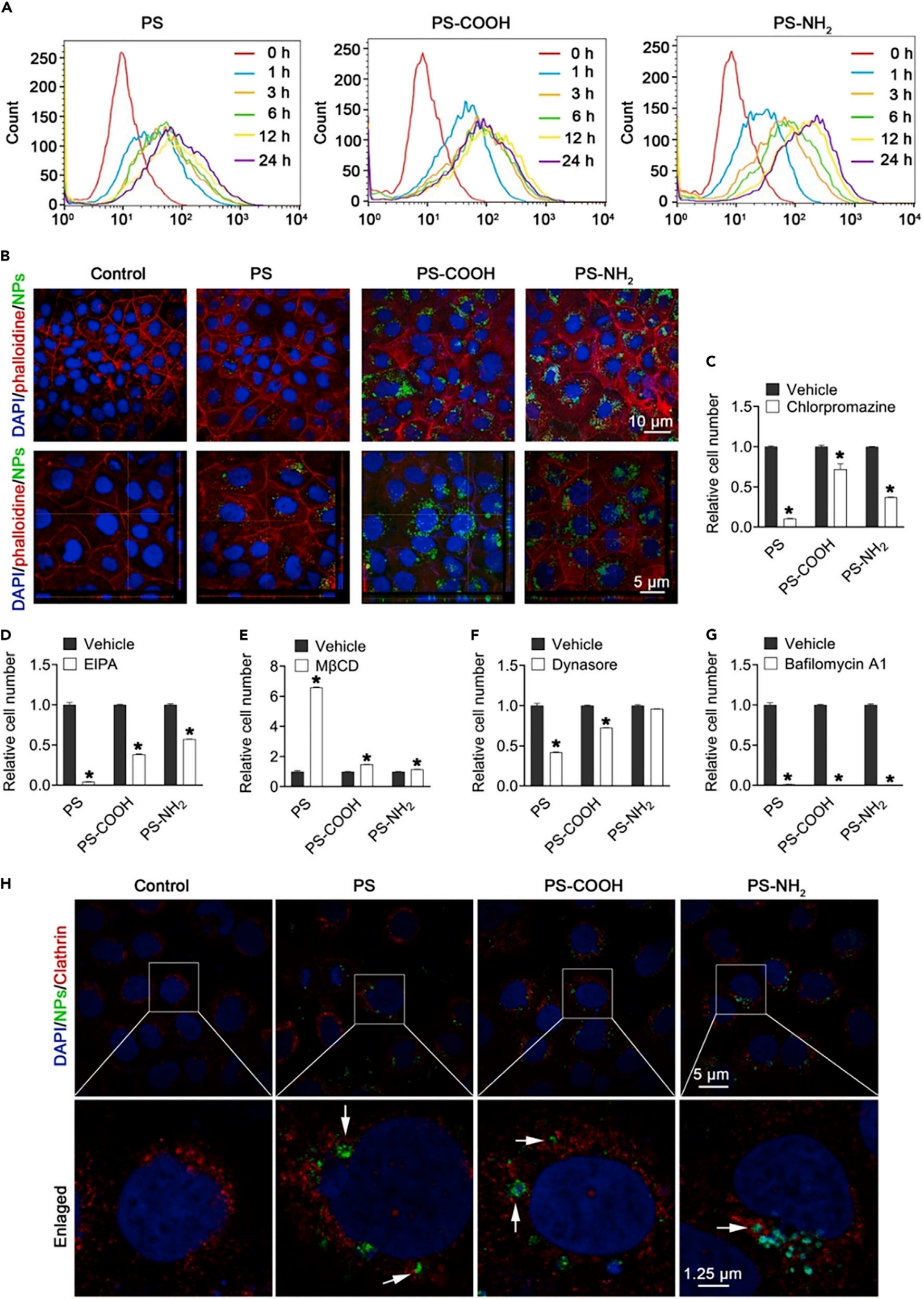
Caco-2 cells internalizing NPLs (A–G) Analysis of NPLs internalization by Caco-2 cells using flow cytometry (A) and Confocal microscopy (B). Flow cytometry analysis of Caco-2 cells pre-treated for 1 h with chlorpromazine (C), EIPA (D), MβCD (E), dynasore (F), and bafilomycin A1 (G) followed by posttreatment with NPLs for 24 h. Localization of NPLs in Clathrin-mediated vesicle examined with confocal microscopy (H). Adopted with permission from Elsevier.^101^

**Figure 3:**
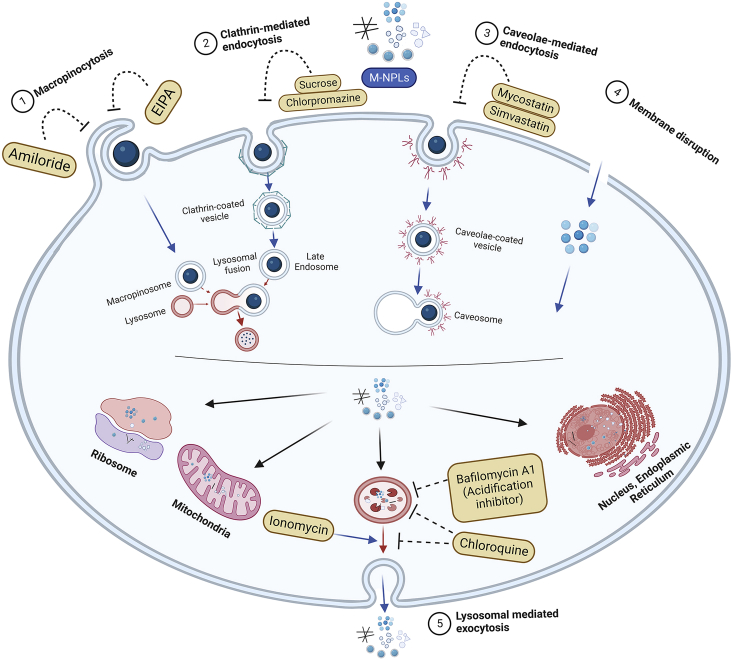
Cellular uptake and release of M-NPLs. M-NPLs utilize various types of endocytosis (1) Macropinocytosis, (2) Clathrin-mediated endocytosis, (3) Caveolae-mediated endocytosis, and disruption of cellular membrane to get internalized. M-NPLs are released from the cells by 4) lysosomal-mediated endocytosis.

M-NPLs are absorbed by endocytosis, as demonstrated by the reception of high-density polyethylene (HDPE) particles via endocytosis and their subsequent transport to the gills.^114^ Xu et al.^101^ investigated the molecular mechanism of NPLs internalization in human intestinal epithelial (Caco-2) cells (Figure 3). They identified macropinocytosis and clathrin-mediated endocytosis as the primary mechanisms for NPLs uptake in Caco-2 cells. A study using the macropinocytosis inhibitor 5-(N-ethyl-N-isopropyl) amiloride (EIPA) to block macropinocytosis found that the uptake of NPLs-COOH, NPLs-NH_2_, and PS-NPLs in Caco-2 cells was reduced. Also, the inhibition of clathrin-mediated endocytosis by the inhibitor chlorpromazine caused a significant reduction in the uptake of NPLs-COOH, NPLs-NH_2_, and PS-NPLs (Figure 2). Moreover, PS-NH_2_ was internalized easily compared to NPLs-COOH and PS-NPLs. Furthermore, dynasore pretreatment significantly reduced NPLs internalization by preventing caveolae and clathrin-mediated endocytosis and, thus, the formation of pinched-off vesicles by limiting dynamin function. Of interest, pretreatment with MβCD, a caveolae-mediated endocytosis inhibitor, significantly increased NPLs internalization by depleting cell membrane cholesterol levels. It is assumed that lowering cholesterol would increase cholesterol-independent clathrin-mediated endocytosis, leading to increased NPLs absorption. Also, bafilomycin A1, an inhibitor that prevents autophagosomes and lysosomes from fusing, completely blocked NPLs uptake.^101^ In addition, NPLs are colocalized with clathrin-related endosomes using confocal immunofluorescence microscopy, implying that NPLs uptake was mediated by clathrin-related endocytosis (Figure 2).^101^

Another study also confirmed respective endocytosis pathways and suggested the involvement of the caveolae-mediated pathway (which was not confirmed by^101^) in M-NPLs internalization. This difference could be due to the use of different caveolae-mediated inhibitors in these studies. Liu et al.^109^ exposed RBL-2H3 cells to PS-*M*-NPLs (50 nm and PS 500 nm, respectively) and used a variety of inhibitors, including sucrose and chlorpromazine (clathrin-mediated pathway inhibitors), simvastatin and mycostatin (caveolae-mediated pathway inhibitors), amiloride (macropinocytosis inhibitors), and chloroquine (endosome acidification and membrane fusion inhibitors). Compared to the control, internalization of PS-NPLs 50 nm was reduced to 46.5% on treatment with sucrose, 60.8% with chlorpromazine, 65.2% with simvastatin, 66.3% with mycostatin, 81.6% with amiloride, and 83.9% when treated with chloroquine. The significant decrease in percentages of internalization caused by sucrose, chlorpromazine, simvastatin, and mycostatin indicates that clathrin and caveolae-mediated endocytosis are the primary pathways for PS-NPLs internalization. In contrast, macropinocytosis is a minor pathway responsible for PS-NPLs internalization for PS-NPLs 50 nm. By using the macropinocytosis inhibitors (amiloride and chloroquine), endocytosis of PS-MPLs 500 nm was decreased to 34.1 and 71.6%, respectively, as compared to the control group, indicating that macropinocytosis is the major pathway for PS-MPLs 500 nm uptake, as previously reported to be responsible for the internalization of larger particles (0.5–1 μm) (Figure 3).^109^

#### Exocytosis

Liu et al.^109^ reported that exocytosis is the primary pathway for the release of M-NPLs from the cells. After internalization, PS-MNPLs accumulated in the lysosome. Bafilomycin A1, an inhibitor of lysosome acidification, and ionomycin which supports cell membrane lysosome fusion were used to examine if these internalized particles could be exocytosed via an energy-dependent pathway. Bafilomycin reduced the release of PS-NPLs (50 nm) by 33% and PS-MPLs (500 nm) by 40% compared to the control. Ionomycin increased the exocytosis of PS-NPLs (50 nm) by 125% and PS-MPLs (500 nm) by 148%. Thus, these results suggest that lysosomal-mediated exocytosis is the major release pathway for M-NPLs.^109^ Alternatively, they can get released passively or by disrupting the cellular membrane.

#### Size-dependent internalization of M-NPLs

Size-dependent cellular internalization of MPLs was observed in marine *Platymonas helgolandica var. tsingtaoensis* and *Scrobicularia quadricauda* (freshwater microalgae) treated with 10 mg/L polystyrene microbeads of various sizes (1, 2, 3, 4, and 5 μm).^117^ In *P. helgolandica var. tsingtaoensis*, 1 μm MPLs were detected in 24% of cells, and 2 μm was present in 11.3% of cells. For *S. quadricauda* cells, the percentages were 43.3% and 15.3% for 1 and 2 μm MPLs, respectively. Confocal laser scanning and 3D image analysis showed no cellular uptake of 3–5 μm of MPLs in these species after 72 h of exposure, demonstrating a size-dependent cellular uptake.^117^ Furthermore, PS 50 and PS 500 nm have shown significant penetration and distribution in lipid membrane compared to PS 5 μm, suggesting size-dependent internalization in the model cell membrane. These particles will likely penetrate the membrane passively through an energy-free pathway.^109^ It is also worth noting that microplastic exposure in the environment is often a heterogeneous mix of different sizes; hence, further studies of possible combinatorial effects of microplastics in terms of different sizes could help researchers better understand potential exposure hazards. However, all current studies suggest that M-NPLs of smaller size are more easily taken up by cells than the larger ones.

## Toxicity of microplastics and nanoplastics

### *In vitro* toxic effects

Accumulating research suggests the toxicological effects of MPLs on different human cells (Table 3).^102^ For instance, after 48 h of treatment, 500 nm PS-NPLs at 50 and 100 μg/mL concentrations reduced HUVEC cell viability to less than 80% of the control.^144^ Moreover, when trypan blue exclusion assay was used to evaluate the cytotoxicity of PS-MPLs in HEK293 cells, the proportion of viable HEK293 cells was significantly reduced after 24 h of exposure to PS-MPLs at 30 and 300 ng/mL concentrations. In addition, the untreated HEK293 cells were homogeneously distributed and examined under a light microscope on the culture dish. Compared to untreated cells, PS-MPLs treated-cells displayed a change in morphology, appearing round and shrinking, and detached from the substrate.^107^ Another study has reported a decrease in the viability of Caco-2 cells after 12 h exposure to PS-NH_2_ (60 μg/mL or higher). Provided the possibility of low-dose exposure of human beings to NPLs in the environment, PS-NH_2_ and PS-COOH have been found cytotoxic to Caco-2 cells at a concentration as low as 30 μg/mL after 48 h.^101^ Moreover, CCK-8 assay has been used to analyze the viability of GES-1 cells in a 48-h exposure to PS-MPLs, L-Cl_2_-PS-MPLs (low dose of chlorinated MPLs), and H-Cl_2_-PS-MPLs (high dose of chlorinated MPLs) at concentrations 1, 10, 20, 50, and 100 mg/L. At low concentrations (1 mg/L), both PS-MPLs and chlorinated PS-MPLs had negligible effects on cell viability. However, the viability of GES-1 cells was significantly reduced following exposure to high concentrations (100 mg/L). Furthermore, there were substantial differences in the cell viability of PS-MPLs and the chlorinated PS-MPLs groups at the same concentrations. For example, when GES-1 cells were exposed to 100 mg/L of pristine PS-MPLs, their viability was 83.9%, compared to 73 and 63.6%, respectively, when exposed to the same concentration of L-Cl_2_-PS-MPLs and H-Cl_2_-PS-MPLs. The findings show that chlorinated PS-MPLs were more harmful to GES-1 cells at high concentrations than PS-MPLs.^108^ Also, a change in the cellular morphology of GES-1 cells from fusiform to round shape in chlorinated PS-MPLs was observed, indicating cellular toxicity. These effects were attributed to the interaction of CL-PS-MPLs with cytoskeleton proteins.^108^

**Table 3:** Toxicity of M-NPLs

M-NPLs (Size)	StudyType/cells/species	Results	Reference
PS-NPs (100 & 500 nm)	*In vitro*, Human Umbilical Vein Epithelial cells (HUVECs)	• Cell membrane damage	Lu, et al., 2022^100^
PS-NPLs, PS-COOH, PS-NH_2_ (100 nm)	Both *in vitro* and *in vivo*, Human intestinal epithelial cells (Caco-2) and Specific pathogen-free (SPF) BALB/c mice (Male, 6 weeks)	• NPLs could bring about hematological system injury and lipid metabolism disorder in mice • PS-NH_2_ were more toxic as compared to PS NPLs	Xu, et al., 2021^101^
PS-MPLs (0.5 μm)	Both *in vitro* and *in vivo*, Granulosa cells and Female Wistar rats, 6 weeks old, weighing ∼180 g	• Number and volume of growing follicles decreased significantly after 1.5 mg/d treatment • Decreases in Anti-Mullerian Hormone at 0.15 and 1.5 mg/d PS-MPs • Increase in MDA and decrease in CAT, SOD and GSH at 0.15 and 1.5 mg/d PS-MPs • Increase in Granulosa cells ROS at 5 and 25 μg/mL PS-MPs and decrease in ROS at NAC +25 μg/mL PS-MPs • Increase in staining and IOD values of newly formed collagen and fibronectin, Bax, and decreases in Bcl-2 at 1.5 mg/d of MPLs • Increase in apoptotic rate in Granulosa cells at μg/mL PS-MPs and decreases in the apoptotic rate at NAC +25 μg/mL PS-MPs • Increases in Wnt/β-catenin signaling and fibrosis	An et al., 2021^102^
PS-NPLs (24 and 27 nm)	*In vivo*, Crucian carp (*Carassius carassius*)	• Feeding and shoaling behavior defects • Defective metabolism • Changes in the appearance and weight of the brain	Mattsson et al., 2015^156^
Green, fluorescent PS-NPLs (25nm)	*In vivo*, Zebrafish (*Danio rerio*)	• Glucose homeostasis disruption • Cortisol elevation and hyperactivity	Brun et al., 2019^157^
PS-NPLs (50 nm), PS-MPLs (10 μm)	*In vivo*, (*Danio rerio*) Larvae	• Increase in ROS with corresponding changes in GSH and antioxidant enzyme activities	Choi, et al.,2020^158^
MPLs and phenanthrene (Phe)-loaded low-density polyethylene (LDPE), <60 μm	*In vivo*, African catfish (*Clarias gariepinus*)	• Liver and gill histopathological changes • Changes in blood biochemistry • Changes in the expression of reproductive axis genes	Karami et al., 2016^159^
Polystyrene Microsphere (70 nm, 5 μm, and 20 μm)	*In vivo*, Zebrafish (*Danio rerio*)	• Liver inflammation and lipid accumulation • Increase in antioxidant enzymes • Variations in liver metabolomics	Lu et al., 2016^104^
Green, fluorescent PS-MPLs (0.1 and 1 μm)	Both *in vitro* and *in vivo*, Human Liver cell lines (HL7702) and SPF male C57 mice	• PS-MPLs induced mitochondrial and nuclear DNA damage • Inflammation and liver fibrosis as results of cGAS/STING pathway activation	Shen et al., 2022^105^
PS-NPLs (20 nm)	*In vivo*, Zebrafish embryo (*Danio rerio*)	• PS-NPLs induced DNA damage in brain tissue • Elevated level of ROS and induced apoptosis in the brain • Yolk exposure to PS-NPLs could cause body malformation	Sökmen et al., 2020^106^
PS-MPLs (3.54 ± 0.39 μm)	*In vitro* Human embryonic kidney 293 (HEK293)	• Antioxidant enzymes were inhibited, which led to ROS-induced cytotoxicity • PS-MPLs induced apoptosis and autophagy • PS-MPLs caused inhibition of NLRP-3 hence diminishing inflammatory response	Chen et al., 2022^107^
PS-MPLs (213.7 ± 8.2 nm)	*In vitro*, Human gastric epithelial (GES-1) cells	• Chlorine disinfection altered PS-MPLs • Chlorinated MPLs altered the morphology of GES-1 cells • PS-MPLs induced mitochondria-dependent apoptosis	Qin et al., 2022^108^
Rhodamine-labeled polystyrene beads (20 nm)	*In vivo*, Time-pregnant Sprague-Dawley rats	• Decrease in weight of fetal and placental weight after 24 h of maternal exposure	Fournier et al., 2020^110^
PS M-NPLs (50 nm, 100 nm and 1 μm)	Both *in vitro* and *in vivo*, Hemocytes and *Mytilus galloprovincialis*	• Motility and immune function of hemocytes were impaired after exposure to PS M-NPLs	Sendra et al., 2020^111^
PS M-NPLs ((0.5μm, 4μm, 10μm)	Both *in vitro* and *in vivo*, Germ cells (GC), Leydig cells (LC), and Sertoli cells (SC) and Male BALB/C mice	• Sperm quality and testosterone levels declined • Inflammation in the testis and disruption of the blood-testis barrier (BTB)	Jin et al., 2021^112^
PS-MPLs (5 μm)	*In vivo*, ICR (Institute of Cancer Research) male mice	• PS-MPLs affected sperm quality • PS-MPLs activated NF-κB pathway • PS-MPLs resulted in abnormal testicular spermatogenesis by inducing inflammation	Hou et al., 2021^160^
High-density polyethylene (HDPE) particles (0–80 μm)	*In vivo*, blue mussel (*Mytilus edulis L*.)	• Histological changes and strong inflammation were observed	von Moos et al., 2012^114^
PS-NPLs (100, 200, 500, 1000nm) & Negative charged PS-NPLs (100, 500, 1000 nm)	*In vitro*, Human induced pluripotent stem cells (hiPSCs)	• Cell viability and self-renewal capacity were decreased • PS-NPLs did not cause chronic toxicity after 14 days	Jeong et al., 2022^115^
PS-NPLs (50 nm)	*In vitro*, Human intestinal epithelial cells (Caco-2)	• PS-NPLs induced structural changes in the nucleus • Minor changes in genotoxicity biomarkers were observed	Domenech et al., 2021^116^
Low-density polyethylene (LDPE) microplastics (11–13 μm)	*In vivo*, Clam (*Scrobicularia plana*)	• LDPE-MPLs induced DNA damage • Time- and tissue-dependent oxidative stress • A significant increase in SOD and CAT activity • Increase in GPx and tissue-dependent GST response • Increase in ROS level • Overall mechanical injury of gills was observed	O'Donovan et al., 2018^151^
PS-NPLs (102.8 ± 4.5 nm)	*In vivo*, *Caenorhabditis elegans*	• Increased expression of PMk-1 • Activation of p38 MAPK pathways • Protective response to PS-NPLs is observed	Qu et al., 2019^153^
PS-NPLs (−)	*In vitro*, Bronchus epithelial (BEAS-2B)	• PS-NPLs caused metabolic changes related to ER stress and autophagy • PS-NPLs also caused autophagy regulation	Lim et al., 2019^154^
PS-MPLs (1, 2, 3, 4 and 5 μm)	*In vivo*, *P. helgolandica var. tsingtaoensis* and *S. quadricauda*	• PS-MPLs (1-2 μm) resulted in a decrease in algal density and affected photosynthesis	Chen et al., 2020^117^
PS M-NPLs (460 nm, 1, 3, 10, 40, & 100 μm)	*In vitro*, Human Dermal Fibroblasts (HDFs), Human Peripheral Blood Mononuclear Cells (PBMCs), Red blood cells (RBCs) & the Human Mast Cell line (HMC-1)	• PS M-NPLs (500 μg/ml, 10-100 μm) were not toxic to human cell lines • RBCs were impacted by both 460 nm and 1 μm PS M-NPLs • Attachment of PS M-NPLs to RBCs caused hemolysis	Hwang et al., 2020^118^
Polymethylmethacrylate (PMMA)-NPLs (∼45 nm)	*In vivo*, *Dicentrarchus labrax*	• NPLs particles caused an increase in mRNA transcripts related to lipid metabolism • A decrease in plasma esterase activity indicates a compromised immune system • Alkaline phosphate level was decreased	Brandts et al., 2018^161^
PS-MPLs (500 nm)	*In vivo*, whiteleg shrimp (*Litopenaeus vannamei*)	• MPLs hampered gonadal development • MPLs suppressed metabolism and limited energy availability • Upregulating the content of gonadal development-related hormones (GIH and MIH) as well as the expression of regulatory hormone genes (GIH, MIH, and CHH) • Co-exposure to MPLs and BPA was more toxic to gonads	Han et al., 2022^162^
Polyethylene Flakes (<400 nm)	*In vivo*, *Hydra attenuata*	• Polyethylene flakes significantly reduced feeding • Reproduction was not affected • Environmental relevant concentration did not show any effect	Murphy et al., 2018^163^
PS-NPLs (70 nm)	*In vivo*, *Daphnia magna* and *Scenedesmus obliquus* (Algae)	• PS-NPLs impacted the concentration of chlorophyll in algae and slowed down population growth • *Daphnia magna’s* body size and reproduction were negatively impacted • The body size and number of neonates also decreased	Besseling et al., 2014^164^
MPLs fragments	*In vivo*, Earthworms (*Eisenia andrei*) and springtails (*Folsomia candida*)	• Polypropylene generated from face mask were found in soil invertebrates • Reproduction and growth in springtail were affected • The springtail was not negatively impacted in terms of survival, esterase activity, oxidative stress, or light avoidance behavior. • Intracellular esterase activity and spermatogenesis in earth worm was affected	Kwak and An, 2021^92^
PS-MPLs (0.5 μm)	*In vivo*, Male Wistar rats	• PS- MPL damaged seminiferous tubule and caused apoptosis of spermatogenic cells • Decreased sperm concentration and motility • PS-MPLs caused oxidative stress and activated p38 MAPK pathway and hence reducing the level of NRF2 • Expression of blood-testis barrier-related protein was also reduced • Overall, the PS-MPLs caused reproductive toxicity	Li et al., 2021^165^
PS-MPLs (0.5 μm)	*In vivo*, Male Wistar rats	• PS-MPLs raised the serum levels of troponin I and creatine kinase-MB (CK-MB) • Caused structural damage and apoptosis in the myocardium, leading to collagen proliferation in the heart • PS M-NPLs cause oxidative stress hence inducing Wnt/β-catenin signaling fibrosis	Li, et al., 2020^166^
PS-MPLs (1–10 μm and 50–100 μm)	Both *in vitro* and *in vivo*, Mouse myoblasts cell line (C2C12) and C57BL/6 male mice	• Muscle fiber regeneration was impaired • Disruption of the equilibrium between myogenic and audiogenic differentiation • Overproduction of ROS in satellite cells was observed • p38 MAPK and NF-κB activation was changed during muscle regeneration	Shengchen et al., 2021^167^
PS-MPLs (100 nm, 5 μm, and 200 μm)	*In vivo*, zebrafish (*Danio rerio*)	• PS-MPLs induced changes in intestinal microbiota • Dysfunction of Intestinal immune cells were also observed • 100 nm PSM-NPLs disrupted gene expression involving phagocyte-produced ROS generation • 100 nm PSM-NPLs also resulted in increased secretion of mucus form secretory cells • 5 μm particles changed lysosome, and 200 μm changed cell surface receptors	Gu et al., 2020^168^
PS NPLs (50 nm)	*In vivo*, Zebrafish (*Danio rerio*)	• Locomotion of the larvae was inhibited • Acetylcholinesterase activity was inhibited • Inhibited acetylcholinesterase activity • Cytoskeleton markers were upregulated	Chen et al., 2017^169^
Low-density polyethylene (LDPE)-MPLs (50–500 μm)	*In vivo*, Catfish (*Clarias gariepinus*)	• The opercular beat frequency of the fishes were increased • The swimming speed of the fishes were reduced due to this accumulation	Tongo et al., 2022^119^
PS-MPLs micro and nanospheres (0.5 and 50 μm)	*In vivo*, ICR (Institute of Cancer Research)	• PS-MPLs caused Mucin decrease in mice’s gut • Dysbiosis in the gut microbiota • PS-MPLs also cause hepatic lipid metabolism disorder	Lu et al.,2018^170^
PS-NPLs (44 and 100 nm)	*In vitro*, Gastric adenocarcinoma (AGS) cells	• PS-NPLs affected the viability of cells, expression of inflammatory genes, and morphology of the cell • PS-NPLs (44 nm) increased the expression of IL-6 and IL-8 genes	Forte et al., 2016^120^

However, in another study, M-NPLs did not significantly reduce cell viability, which can be attributed to their low concentration and dosage, as various concentrations between 50 μg/L and 10 mg/L of MPLs (polyethylene and polystyrene) did not affect cerebral (T98G) and HeLa (epithelial) cell viability.^144^ Moreover, different sizes (100 nm or 500 nm) of PS-NPLs were not observed to reduce human umbilical vein endothelial cells (HUVECs) viability after 24 h of exposure.^144^ Human intestinal epithelial (Caco-2) cells, when exposed to PS and PS-COOH and nanoplastics at a dose of 60 μg/mL or higher, did not show altered cell viability.^101^ Jeong et al.^115^ demonstrated the accumulation of NPLs inside hiPSCs cells, suggesting the potential threat of maternal exposure to plastic particles that might interfere with human embryogenesis. Using hiPSCs’ ability to self-renew, cells exposed to PS-NPLs were studied for up to 14 days while undergoing EB formation and neuronal differentiation. Although there were no significant effects on hiPSC differentiation potential *in vitro*, the large-scale intracellular accumulation of NPLs (up to 1000 nm diameter) in hiPSCs suggests the possibility of causing unexpected alterations in human embryogenesis, where sophisticated differentiation processes co-occur. This notion is supported by previous research demonstrating evidence of NPLs translocation from maternal to fetal tissues through the placenta.^110^

### *In vivo* toxic effects of microplastics and nanoplastics on organ systems

There exists an abundance of research suggesting the accumulation of M-NPLs in several vital organs of the body and their negative effects on these organs (Table 3).^171^ M-NPLs can also transport microorganisms and contaminants^36^^,^^98^^,^^118^ and release chemicals from their matrixes.^172^ Cytotoxic effects of M-NPLs are attributed to prolonged exposure of the body/tissue to these particles as they persist in the body and translocate to other cells/organs.^30^^,^^113^^,^^173^^,^^174^ While inside the body, M-NPLs affect a wide range of biological processes resulting in the generation of free radical species, ROS, and cytokines, causing cellular damage, inflammatory/immune responses, DNA damage, and neurotoxic and metabolic manifestations.^175^^,^^176^ These adverse effects are primarily determined by the individual’s level of exposure and susceptibility.^134^ These particles spread from the exposure site to other tissues^113^ and remain intact in the body, becoming a constant source of exposure to the various human organ systems.^173^ PS-NH_2_ treatment has been reported to reduce net body weight gain, absolute large intestine weight, and testicular weight in mice.^102^ In addition, white blood cells (WBCs) count and total plasma cholesterol levels were also reduced, whereas platelet (PLT) levels were increased.^101^ These particles also affect net body metabolism by interfering with metabolic enzymes and disrupting energy balance.^177^ For instance, NPLs have also been found to interfere with the mobilization of energy reserves by inducing lipid metabolism.^161^

The primary organs where M-NPLs accumulate include the brain, heart, liver, kidney, lungs, and placenta.^105^^,^^127^M-NPLs were assessed using Raman Micro-spectroscopy in physiologically pregnant women, and a total of four placentas contained 12 microplastic fragments (5–10 μm) having spherical or irregular shapes. M-NPLs were discovered on the fetal side in five cases, whereas four points presented them on the maternal side and three on the chorio-amniotic membranes, respectively. The plastic particles detected in them are used in personal care products, cosmetics, finger paints, paint and coatings, and adhesives, among other things. Endocrine-disrupting chemicals were suspected to be carried by these MPLs, which can have long-term effects on human health.^127^ Xu et al.^101^ treated 6-week-old Specific pathogen-free (SPF) BALB/c male mice with 100μL PS, PS-COOH, and PS-NH_2_ (10 mg/mL) by gavage once a day (1 mg/day). For histopathological examination, tissues (brain, liver, kidney, lungs, ileum, colon, and testis) of the treated mice were fixed in 4% paraformaldehyde solution for 12 h and then embedded in paraffin before being cut into 5 μm sections. This investigation revealed that NPLs accumulate and affect almost all major organs of the body.^101^ The toxicity of M-NPLs in major organ systems is discussed in detail below.

### Nervous system toxicity and neurodegenerative effects of microplastics and nanoplastics

Several studies have found that M-NPLs cause neurotoxicity. NPLs have been shown to cause abnormal layering of neurons and abnormal neuronal characteristics in the brain’s cerebral cortex characterized by nuclear pyknosis. In mice brain tissues exposed to PS-NH_2_, immunohistochemical results confirmed increased caspase-3 signals, indicating neuronal cell apoptosis. Also, the levels of cytokines (TNF-α and IL-6) were upregulated in the brain, suggesting cytokine-induced inflammation in these tissues.^101^ Moreover, MPLs administration to European seabass decreased acetylcholinesterase (AChE) enzyme release, initiated oxidative stress and lipid peroxidation, and induced anaerobic energy production pathways leading to abnormal swimming behavior.^177^ Neural cells treated with these particles caused toxicity and decreased metabolic rate.^177^ These effects of M-NPLs could be attributed to the accumulation of activated immune cells in the brain, oxidative stress, and increased circulatory pro-inflammatory cytokines (TNF-α and IL-6).^101^^,^^178^ Other studies have also demonstrated the toxic effects of these plastic particles on the brain.^179^^,^^180^ For example, O'Donovan et al.^151^ reported that LDPE MPLs in clam cause neurotoxicity because of changes in acetylcholinesterase (AChE) activity or by reaching the brain leading to oxidative stress, which damages cells causing neurodegenerative and neurodevelopmental problems.^181^

### Microplastics and nanoplastics' impact on the endocrine system

M-NPLs can also either affect the endocrine cells or interfere with the interplay of hormones, affecting the endocrine system. PS-MPLs have been observed to affect Anti-Mullerian hormone (AMH) levels by inducing apoptosis and fibrosis in granulosa cells and rats’ ovaries, respectively, through oxidative stress.^102^ M-NPLs also act as endocrine-disrupting chemicals (EDCs) or help transport EDCs to the body of an organism by adsorbing these EDCs.^182^ Adsorption and desorption of common steroid hormones such as 17-estradiol (E2) and 17-ethynylestradiol (EE2) on microplastics in coastal water revealed that E2/EE2 had a desorption capacity of over 40% of its adsorption capacity on microplastics.^182^ MPLs also impede hormonal control of reproduction via anti-oxidative stress response, sex hormone changes, and disrupted transcription of steroidogenic genes in the reproductive axis. Transcriptome analysis also revealed that exposure to 20 μg/L MPLs significantly impacted steroid hormone production and cytochrome P450 pathways in fish testes.^183^ MPLs were also shown to block gonadal development in *Litopenaeus vannamei* (white-leg shrimp) by upregulating the gonadal development-related hormones (GIH and MIH) levels as well as the expression of their regulatory hormone genes (GIH, MIH, and CHH).^162^

### Toxicological effects of microplastics and nanoplastics on the reproductive system

MPLs and NPLs have been found to affect both the morphology and physiology of the reproductive system in *Hydra attenuata*,^163^ mice,^101^ *Daphnia magna*,^164^ and other animal species. As previously discussed, An et al.^102^ have demonstrated the role of PS-MPLs in affecting reproduction in rats. Moreover, mice treated with NPLs showed an increased accumulation of immature germ cells in the lumen of seminiferous tubules, causing testicular atrophy.^102^ When oysters were exposed to PS-MPLs, the number of oocytes and sperm velocity decreased significantly,^142^ and a similar decrease in *C. elegans* reproductive potential has also been reported.^184^ Immunohistochemical analyses of mice testicular tissues exposed to PS-NPs, PS-COOOH, and PS-NH_2_ have revealed increased caspase-3 signals, indicating cell apoptosis.^101^ Also, TNF-α and IL-6 were upregulated in mice testis, suggesting cytokine-induced inflammation in these tissues.^102^ The causes of these reproductive effects are attributed to oxidative stress, cytokine increase, and inefficient energy metabolism induced by M-NPLs.^29^^,^^101^ The COVID-19 pandemic has increased face mask pollution, and the release of nanofibers from face masks has been reported to inhibit reproduction and growth.^92^ PS-MPLs exposure also damages the seminiferous tubules, causing apoptosis in spermatogenic cells and lowering sperm motility and concentration, increasing sperm abnormalities.^165^

### Microplastics and nanoplastics damage the skeletal and cytoskeleton system

The physiological integrity of the skeletal and cytoskeletal systems is critical for the normal physiology and morphology of an organism. According to Shengchen et al.,^167^ PS-MPLs cause an increase in ROS, which affects skeletal muscle regeneration by converting myoblasts to adipocytes. PS-MPLs of two sizes, 1–10 and 50–100 μm, were utilized to test their influence on mice’s anterior tibial (TA) muscle growth and healing after injury. PS-MP exposure slowed skeletal muscle regeneration (inversely linked with particle size) and reduced muscle fiber diameter and the cross-sectional area (CSA). However, PS-MPLs administration did not impact myoblast cell viability. Nonetheless, it increased intracellular ROS formation and oxidative stress, hence limiting myogenic development by lowering the p38 mitogen-activated protein kinase (MAPK) signaling pathway, phosphorylation and encouraging adipogenic differentiation by elevating Nuclear Factor Kappa B (NF-κB) expression, which was mitigated by N-acetyl cysteine (NAC).^167^ Micro- and macroplastics have also been found to reduce the skeletal growth rate, affecting the growth, feeding, and behavior of *Lophelia pertusa* (cold-water coral).^185^ Similarly, PLA-MPLs have been shown to accumulate in higher concentrations and inhibit the skeletal development of zebrafish.^34^

A recent study has highlighted the effects of chlorinated PS-MPLs on the cytoskeleton.^108^ Generally, at the earliest stages of cellular motility, perceptive and exploratory cytoskeletal components such as filopodia and microspikes also develop. According to the findings of this study, both structures can be seen in chlorinated PS-MPLs administered to GES-1 cells. These findings suggest that chlorinated PS-MPLs could cause alterations in GES-1 cell shape and cytoskeleton. The cells internalized both non-chlorinated and chlorinated PS-MPLs, but it was discovered that chlorinated PS-MPLs interacted with cytoskeletal proteins, resulting in changes in cellular morphology.^108^

### Involvement of microplastics and nanoplastics in lymphatic and immune system toxicity

M-NPLs have been shown to affect the immune system, as demonstrated by the harmful effects of PS-MPLs (100 nm, 5 μm, and 200 μm), causing intestinal immune cell dysfunction, including phagosome dysfunction and immune system regulation in Zebrafish.^168^ Moreover, an increase in the number of pathogenic bacteria was also observed on PS-MPLs exposure.^168^ However, depending on the spread and host reaction, the toxic effects of M-NPLs might be local or systemic. They may also lead to autoimmune diseases in genetically susceptible individuals,^53^^,^^134^ possibly induced by oxidative stress and increased immune modulators or cell activation, resulting in the development of antibodies against self-antigens.^186^ The roles of M-NPLs in immune system-related diseases such as rheumatic diseases^186^ have also been reported. The size-dependent effects of polypropylene-MPLs on human-derived Peripheral blood mononuclear cells (PBMCs) can cause an increase in histamine, which causes a local immunological response.^187^ In sea urchins, the total coelomocytes have been observed to increase with a higher ratio of red/white amoebocytes.^188^ Moreover, the increase in phenoloxidase activity caused by MPLs ingestion in *Chironomus riparius* larvae shows that immunological responses can serve as a sensitive predictor of the sub-lethal consequences of MPLs ingestion.^189^ Furthermore, effects of NPLs on hemocyte count, hemolymph, and neutrophil function are reported in *D. magna*, *Mytilus galloprovincialis*, *Pimephales promelas*, and other species.^161^^,^^190^^,^^191^ Additional studies have reported MPLs affecting neutrophil function, leukocytes, and cellular innate immune parameters.^161^^,^^192^^,^^193^^,^^194^

### Exposure to microplastics and nanoplastics is linked to cardiovascular disease

The cardiovascular system is the principal transport mechanism regulating blood circulation, which is why it is highly susceptible to M-NPLs toxicity; for instance, a decreased heart rate was observed in *Oryzias melastigma* onM-NPLs exposure and even depicted a *trans*-generational trend.^183^ M-NPLs can induce oxidative stress and internalization by cardiac sarcomeres, and the subsequent apoptosis might be responsible for arrhythmic heart functionality.^169^^,^^195^^,^^196^ The imbalance of ROS and antioxidants caused by M-NPLs in the cardiovascular system caused these particles to engage with nitric oxide signaling in endothelial cells, converting nitric oxide to cytotoxic peroxynitrate.^166^^,^^197^ This reduces endothelium nitric oxide bioavailability, whereas high peroxynitrite levels are cytotoxic, damaging cells' DNA, proteins, and lipids.^198^ The highest concentration of NPLs in the cardiovascular system has been reported in the pericardial sac,^199^ and embryos treated with 700 nm PS plastic particles showed their uptake through the circulatory system and accumulation in the pericardium.^200^ M-NPLs have been observed to induce hemolysis and venous and arterial thrombosis through van der Waals interactions.^118^^,^^201^^,^^202^^,^^203^ Thrombosis can lead to ischemic stroke and pulmonary embolism.^204^^,^^205^ An increase in the expression of P-selectin on the platelet surface was considered one of the reasons responsible for platelet aggregation leading to thrombosis.^206^ A subsequent decrease in thrombin because of the attachment of factors VII and IX (human coagulation factors) to PS-NPLs might also play a role.^207^

### The impact of microplastics and nanoplastics on the respiratory system

M-NPLs are inhaled from the environment and deposited in human lungs, causing respiratory system toxicity. Using FTIR spectroscopy,^61^ identified 39 different MPLs in 11 of 13 human lung tissue samples, with an average of 1.42 ± 1.50 MPLs/g of tissue. PP (23%), PET (18%), and resin (15%) were the most abundant polymers found. These MPLs were discovered to accumulate in all areas of human lung samples, including the upper, middle/lingular, and lower regions.^61^ This suggests that inhaling MPLs from the environment may contribute to MPLs accumulation and, thus, toxic effects on the respiratory system. A similar study found polymeric particles (less than 5.5 μm) and fibers (8.12–16.8 μm) in 13 out of 20 autopsies of lung tissue samples, with polyethylene and polypropylene as the dominant polymers.^83^ MPLs have also been found in 21 different types of human sputum, with polyurethane dominating, followed by polyester, chlorinated polyethylene, and alkyl varnish accounting for 78.36% of the total MPLs. Most aspirated MPLs discovered are less than 500 μm in size.^84^ According to,^208^ MPLs may affect lung surfactant (LS), known for lowering surface tension and making breathing easier by preventing alveoli from collapsing after exhalation. The presence of PS-MPLs changes the phase behavior, surface tension, and membrane structure of the LS. In a hybrid system of polystyrene and LS, the adsorption of phospholipid components by polystyrene was significantly higher than that of proteins. Furthermore, polystyrene can speed up the conversion of ascorbic acid to deoxy-ascorbic acid, creating hydrogen peroxide (HO_2_O_2_) in simulated lung fluid (including LS) and increasing the concentration of hydroxyl radicals (⋅OH).^208^ NPLs have also been reported to influence lung physiology and morphology in mice, thickening alveolar walls and causing pulmonary interstitial fibrosis.^101^ TNF-α and IL-6 levels were also elevated in the lungs of mice, implying that these tissues are prone to cytokine-induced inflammation.^101^ In fish, MPLs administration caused an increase in opercular respiratory rate,^209^^,^^210^ a viable alternative in assessing stress and aerobic metabolism^119^ and linked with oxygen consumption. This evidence demonstrates the presence and toxic effects of M-NPLs in respiratory systems.

### Microplastics and nanoplastics' effects on the digestive, excretory, and urinary systems

M-NPLs impact all components of the digestive, excretory, and urinary systems. Schwabl et al.^128^ conducted a prospective case series with eight healthy volunteers (ages 33 to 65) to see if M-NPLs could be detected in their stool samples. All samples contained a median of 20 MPLs (50–500 μm) per 10 g of human stool. Nine different types of plastics were found, with polypropylene and polyethylene terephthalate being the most common.^128^ This suggests inadvertent ingestion of MPLs from various sources.

One of the essential parts of the digestive and excretory system, the liver, is affected by MPLs and NPLs. Mice liver treated with NPLs showed immune cell infiltration, vacuolization of hepatocytes with lateral nuclei, shrinkage of hepatocytes with pyknotic nuclei, and an increase in sinusoidal gaps.^101^ NPLs also caused crypt dysplasia, and lymphocyte aggregation in the colon, destroying the ileum epithelium and affecting villi.^101^ The presence of M-NPLs in the digestive system triggers a local inflammatory response, damages the intestinal barrier and gut permeability, and impacts the microbiota population.^112^^,^^170^ In the gastric mucosa, PS-MPLs have been shown to modify gene expression, induce the production of IL6, IL8, and IL1β cytokines, and inhibit cell viability.^120^ Kidneys (an essential part of the urinary system),^211^ when exposed to NPLs, resulted in glomerulus shrinkage, renal tubule atrophy, and immune cell accumulation. TNF-α and IL-6 were also upregulated in mice kidneys, suggesting cytokine-induced inflammation in these tissues ^103^Mechanisms of microplastic and nanoplastic toxicity.

#### Oxidative stress and reactive oxygen species generation

**Figure 4:**
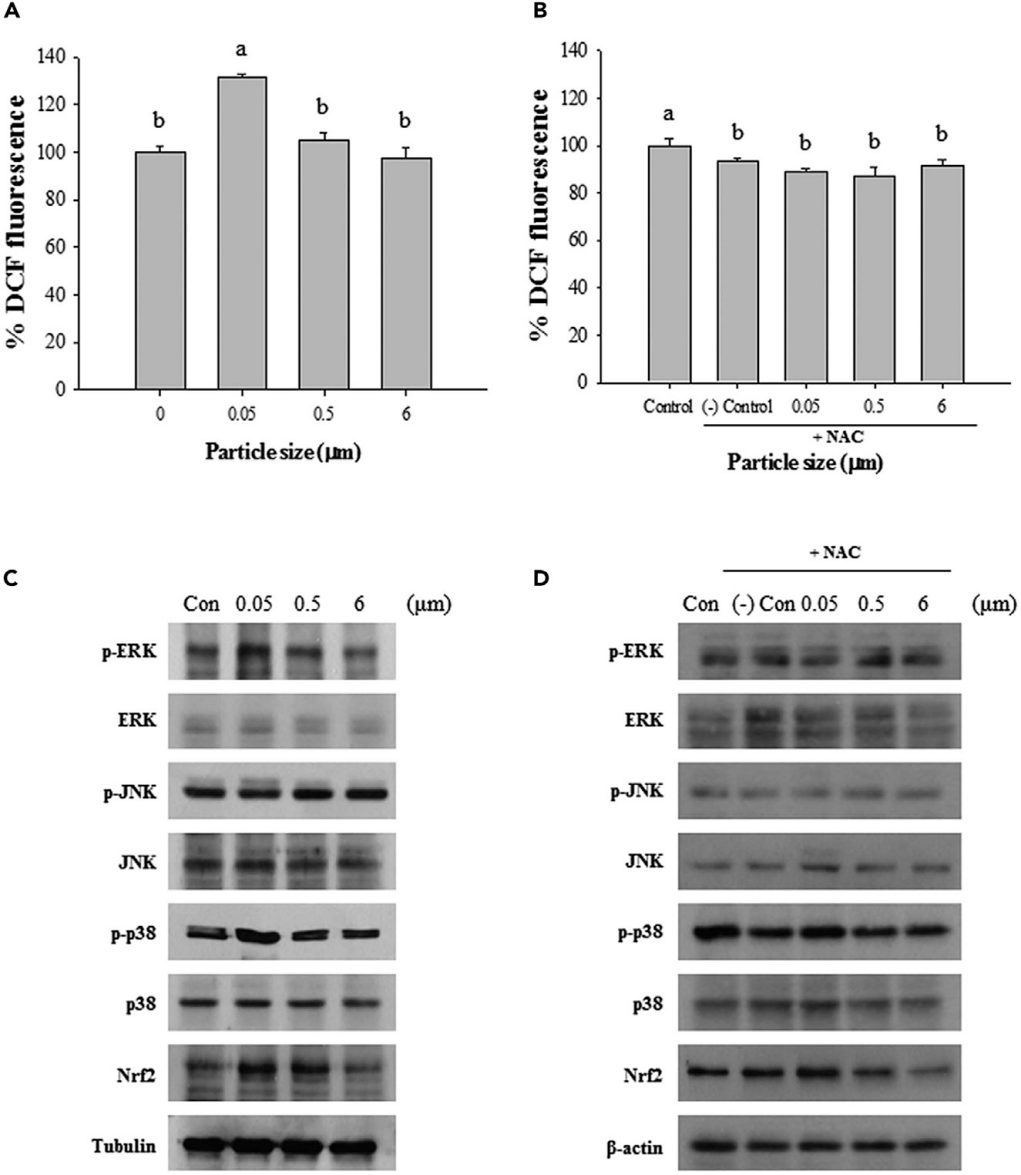
Effects of various polystyrene microbeads on ROS generation, and phosphorylation of MAPK signaling proteins (A–D) ROS Level (A) ROS level with 0.5 mM of NAC administration (B), MAPK protein phosphorylation (C) and MAPK protein phosphorylation with 0.5 mM administration (D). Adopted with permission from ACS Publications.^214^

Reactive oxygen species (ROS) are highly reactive molecules produced by O_2_, and include peroxides, superoxide, hydroxyl radicals, singlet oxygen, and alpha-oxygen.^141^ Oxidative stress is a condition that arises when the body’s cells are overrun with free radicals and ROS. This stress can disrupt components of the cells, such as proteins, and DNA, contributing to a wide range of health issues such as diabetes, cancer, neurological, and cardiovascular illnesses.^212^^,^^213^ MPLs have also been evaluated for their ROS generation potential, releasing oxidizing chemicals and causing inflammation and oxidative stress (Figure 4).^91^^,^^101^^,^^214^^,^^215^ M-NPLs also possess ROS as a byproduct of polymerization and processing, which further increases as these M-NPLs are exposed to degradation.^216^ Trojan horse effects also have been involved in M-NPLs-induced ROS generation and oxidative stress. M-NPLs, through the Trojan horse effect, can cause oxidative stress, producing inflammation and damage.^217^ Adding polycyclic aromatic hydrocarbons (PAHs) to NPLs may amplify the disruption of energy metabolism in mitochondria through the Trojan horse effect.^217^ PS-NPLs are reported to act as carriers of benzo(*a*)pyrene (BaP) mussel hemocytes and produce toxic effects through the Trojan horse effect.^218^ GES-1 cells treated with chlorinated PS-MPLs showed an increase in ROS and a decrease in GSH production compared to the control and PS-MPLs group.^108^ HUVECs cells exposed to 100 nm and 500 nm PS-NPLs did not show an increase in intracellular ROS.^144^ Oxidative stress mediated by M-NPLs has been shown to destroy epithelial cells such as Caco-2, LS174T, and HT-29 cell lines.^219^ Human cerebral and epithelial cells were affected by the high concentration of ROS on administration of 0.05–10 mg per liter of MPLs.^220^ Chen et al.^107^ assessed the redox status of HEK293 cells treated with PS-MPLs (3–300 ng/mL) for 24 h with a 2′,7′-dichlorofluorescein diacetate (DCFH-DA) assay. The expression of the antioxidant enzyme (HO-1) was also measured in this study using western blot assays. Significant oxidative stress was only evidenced in HEK293 cells exposed to PS-MPLs at 300 ng/mL for 24 h, while there was no significant change in HO-1 expression. PS-MPLs have also been shown to boost ROS levels in HEK293 cells by suppressing the HO-1 enzyme. Hence, M-NPLs toxicity can be attributed to oxidative stress and ROS generation.

##### The nuclear factor-erythroid factor 2-related factor 2 (NRF2) signaling pathway

**Figure 5:**
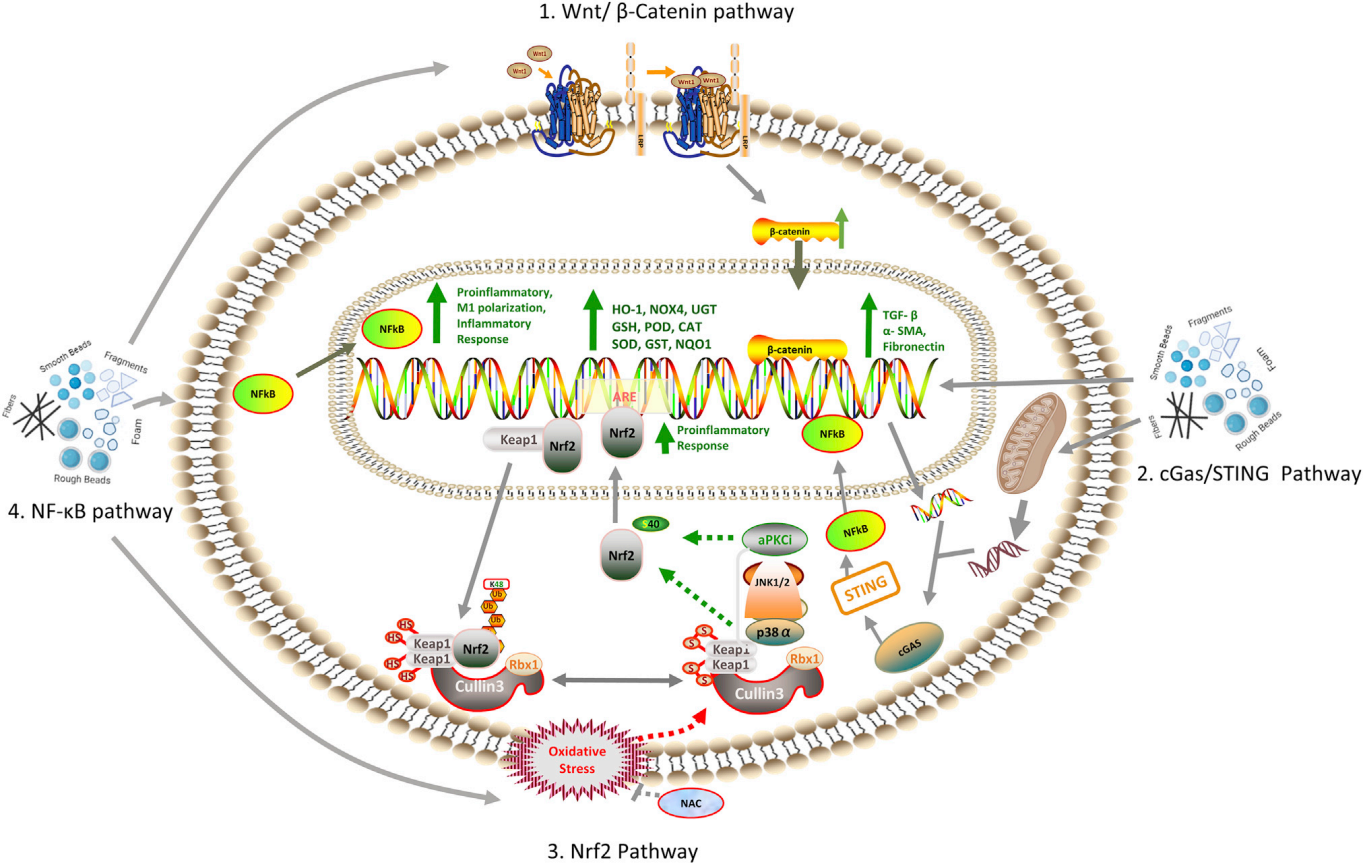
The various molecular pathways utilized by M-NPLs result in toxicity: 1. Wnt/ β-Catenin signaling pathway At the molecular level, when Wnt ligands bind to its cell surface transmembrane receptor, β-catenin will be degraded and then aggregate in the cytoplasm. As β-catenin accumulates to a certain level, it dissociates and possibly undergoes nuclear translocation, thus regulating the expressions of some downstream genes such as α-SMA, fibronectin, and TGF-β, all of which are implicated in fibrogenesis. (1) M-NPLs administration caused an increase in the level of Wnt, β-catenin, p-β-catenin; markers of Wnt/ β-catenin signaling pathway and TGF-β, Collagen I, Collagen III, α-SMA and fibronectin; markers of fibrosis. (2) cGAS/STING Pathway:M-NPLs following interaction with the nucleus and mitochondria causes damage and release of nuclear and mitochondrial DNA fragments into the cytoplasm, which is recognized by the cGAS protein. cGAS protein activates STING protein which in turn causes the internalization of NF-κB. NF-κB causes promote the transcription of pro-inflammatory cytokines. (3) NRF2 signaling pathway:M-NPLs exposure resulted in an increase of intracellular ROS, which caused activation of MAPK proteins (p38 and ERK). These proteins could dissociate Keap-1 from NRF2, resulting in NRF2 translocation into the nucleus and enhanced transcription of antioxidant genes. (4) NF-κB signaling pathway:M-NPLs exposure caused an increase in the level of NFkB protein, which after nuclear translocation, caused an increase in the level of inflammatory, pro-inflammatory cytokines, and M1 polarization.

NRF2 signaling pathway is one of the critical mechanisms utilized by M-NPLs for mediating ROS production and oxidative stress (Figure 5).^221^ M-NPLs, after internalization, have been reported to induce ROS production in marine copepod *Paracyclopina nana*,^214^ causing phosphorylation of MAPK proteins (p38 and ERK).^214^ The antioxidant response is observed owing to activated p38 and ERK, causing detachment of KEAP1 (an inhibitor of NRF2) from NRF2. However, treatment with NAC (ROS scavenger) after MPLs treatment increased ROS level, and activation of p38, ERK, and NRF2 was not observed (Figure 4).^214^ This implies that oxidative stress caused by M-NPLs might trigger oxidative stress-dependent signaling pathways regulated by p-p38, *p*-ERK, and NRF2. Furthermore, the protective effects of hydrogen sulfide (H_2_S) against NPLs toxicity have also been explored. It was discovered that NPLs in the absence of H_2_S limited the accumulation of NRF2 and hence the expression of NRF2-regulated antioxidant genes. H_2_S elevated the expression of HO-1 and NQO1 by facilitating the nuclear accumulation of NRF2.^221^ Yet in another study, p38 MAPK-NRF2 pathway activation in response to MPLs treatment caused rat testicular toxicity involving the activation of p38 MAPK, elevation in p38 phosphorylation, and hence a decrease in NRF2 levels. All the above-mentioned research points to the fact that the NRF2 pathway is a major mediator of M-NPLs toxicity.^165^

##### Wnt/β-catenin signaling pathway

**Figure 6:**
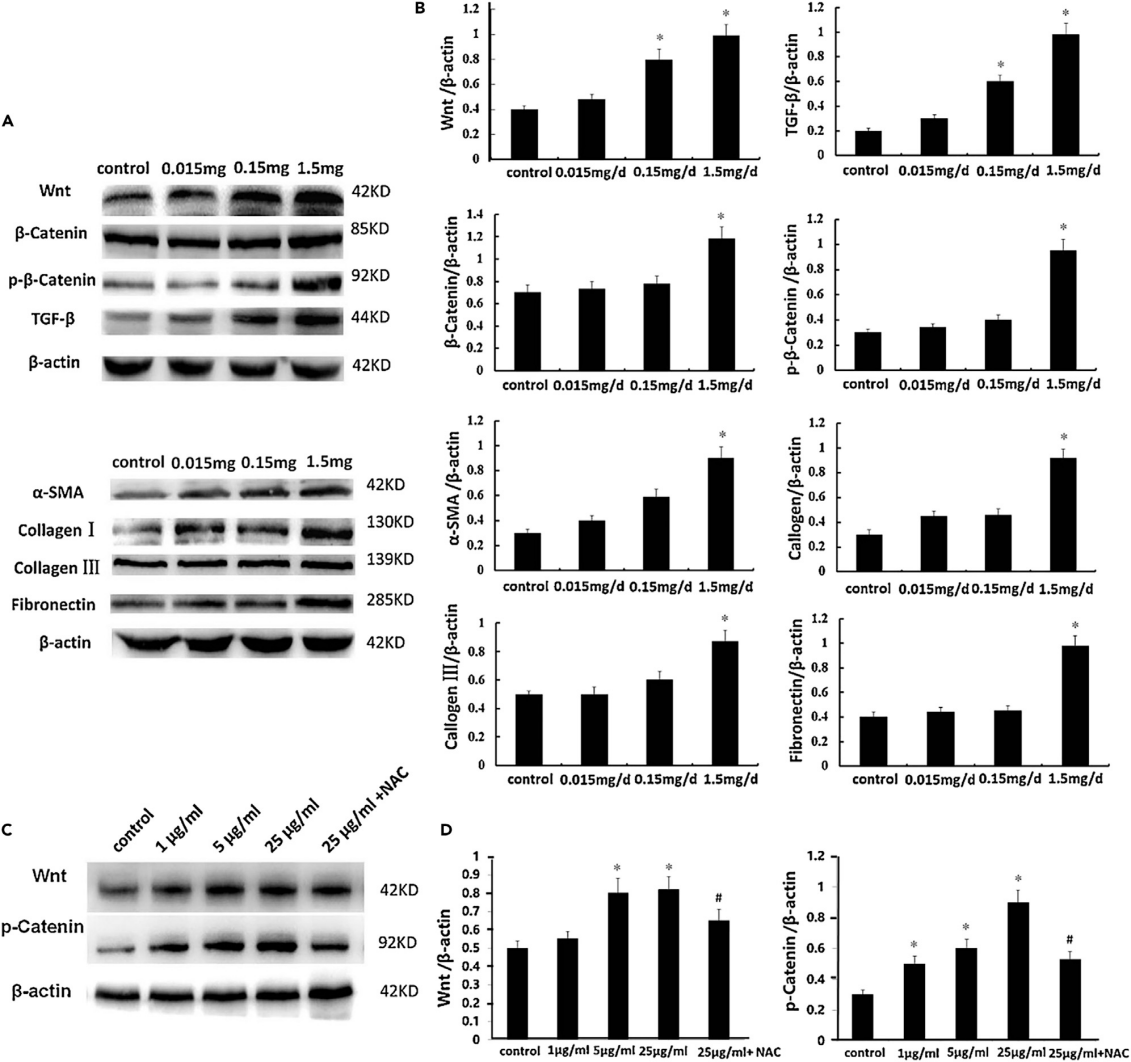
Effects of PS-MPLs on Wnt/β-catenin signaling proteins and the expression of fibrosis markers in GCs and rat ovaries (A–D) Representative gel bands of ovarian fibrosis markers and Wnt/β-catenin signaling pathway proteins (A and C) Densitometric analysis of ovarian fibrosis markers and Wnt/β-catenin signaling pathway proteins in ovarian tissue (B) and GCs (D). Adopted with permission from Elsevier.^102^

The Wnt/β-catenin signaling pathway plays a vital role in maintaining cellular homeostasis and is a highly conserved evolutionarily process. This pathway regulates critical processes, including embryo development, cell proliferation and differentiation, cell death, and cancer.^222^ Wnt, key proteins of the Wnt/ β-Catenin signaling pathway, are lipid-modified glycoproteins that are released and enable cell-to-cell communication, hence regulating processes like cell division, differentiation, and growth.^223^ Another essential protein in this system, β-catenin, performs two functions: it controls and coordinates genes' transcription and regulates cell-cell adhesion.^224^ It is a fact that oxidative stress triggers the Wnt/β-catenin signaling pathway, which is involved in ovarian fibrosis (Figure 5). An et al.^102^ has reported a link between pre-menopausal infertility and environmental pollutants by demonstrating an increase in signaling molecules of Wnt/β-Catenin pathway including Wnt, β-catenin, p-β-catenin (western blotdata) along with an increase in expression of ovarian fibrosis markers after PS-MPLs administration. This suggests that MPLs treatment activates Wnt/β-Catenin signaling components, promoting ovarian fibrosis, as shown in Figure 6.^136^

#### Microplastics and nanoplastics induce inflammation by activating Nuclear Factor Kappa B (NF-κB) and cGas/STING signaling pathways

##### Production of inflammatory cytokines

**Figure 7:**
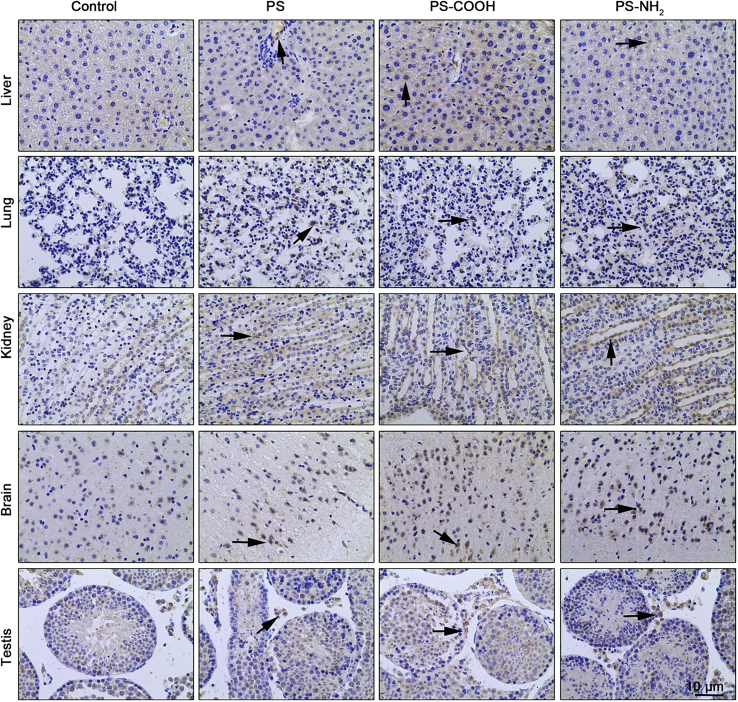
NPLs-induced IL-6 expression in mice tissues The immunohistochemical staining was performed to examine IL-6 expression in the paraffin sections of the tissues shown in the figure, with positive signals indicated by arrows. Adopted with Permission from Elsevier.^101^

M-NPLs have been shown to stimulate the production of inflammatory cytokines, producing local and systemic inflammation (Figure 7).^101^ Liu et al.^225^ studied the effects of 500 μg/L polystyrene microplastics (PS-MPLs) on mice with intestinal immune imbalance and observed an increase in the expression of inflammatory cytokines (TNF-α, IL-1β, and IFN-γ). Also, the formation of granulocytomas in epithelial cells of Blue mussels, which is a non-neoplastic inflammatory cellular response, indicates that environmental pollutants such as HDPE-MPLs can stimulate inflammatory response.^114^ 14-day exposure to 0.5 μm-diameter PS-MPLs has been shown to induce microbiota dysbiosis and inflammation, as demonstrated by the increased mRNA levels of IL1-α, IL-1β, and IFN- γ and their protein levels in the gut of adult zebrafish.^102^ C57BL/6 mice treated with polyethylene-MPLs (6, 60, and 600 μg/day for five consecutive weeks) showed inflammation caused by an increase in Toll-like receptor 4 (TLR4), AP-1, and IRF5 expression in colon and duodenum.^226^ Toll-like receptor 4 is a pattern recognition receptor (PRR) family transmembrane protein that stimulates the innate immune system by activating the intracellular signaling pathway NF-κB and generating inflammatory cytokines when activated/secreted.^227^ Similar results are reported by,^228^ where a 21-day exposure to MPLs caused microbiota dysbiosis and inflammation. As a result of these findings, chronic inflammation and high oxidative stress in the gut have been linked to intestinal microbiota dysbiosis and metabolic diseases.^229^^,^^230^

Moreover, in zebrafish larvae, co-exposure to chlorinated polyfluorinated ether sulfonate (F–53B) and PS-MPLs significantly increased pro-inflammatory cxcl-clc and il-1 gene transcripts and iNOS protein levels. Furthermore, increased protein production of Nuclear Factor Kappa B (NF-kB) coincided with an elevation in the expression of most immune-related genes, implying that the NF-kB pathway is mechanistically implicated in these reactions.^226^ Treatment of GES-1 cells with chlorinated and non-chlorinated PS-MPs showed an increase in IL-1β and IL-6 compared to the control group. Likewise, IL-1β and IL-6 mRNA levels were higher in the chlorinated group than in the non-chlorinated one.^108^ All these studies imply that M-NPLs can induce an inflammatory response.

##### Nuclear Factor Kappa B (NF-κB) signaling

Nuclear factor kappa B (NF-κB) target genes are suggested to play a role in M-NPLs-induced inflammation (Figure 5). NF-κB has been shown to induce pro-inflammatory genes, M1 polarization and increase cytokine production (IL-1, IL-2, IL-6, IL-8, IL-12, and TNF- α.^11^ When mice were exposed to high levels of MPLs, the pro-inflammatory molecule NF-κB, as well as the inflammatory factors interleukin (IL-1 and IL-6, increased significantly, whereas the anti-inflammatory molecule NRF2/HO-1 decreased, implying that abnormal sperm quality in mice, caused by PS-MPLs exposure, is linked to the NRF2/HO-1/NF-κB pathway.^126^ Furthermore, increased NF-κB protein synthesis is correlated with increased expression of most immune-related genes, showing that the NF-κB pathway is mechanistically involved in these events.^231^

##### cGas/STING pathway

M-NPLs also function through the cGas/STING pathway (Figure 5). The stimulator of interferon genes (STING), also known as transmembrane protein 173 (TMEM173), is encoded by the STING1 gene.^232^ Cyclic GMP-AMP synthase (cGAS) is a component of the cGAS-STING DNA sensing pathway,^233^ binding to microbial DNA and self-DNA that enters the cytoplasm and catalyzes cGAS production. It subsequently functions as a second messenger, binding to and activating the ER protein STING, which triggers the production of type-I IFNs.^234^^,^^235^ Shen et al.^105^ reported that PS-MPLs cause inflammation and liver fibrosis by activating the cGas/STING pathway. When mouse liver cells were co-cultured with PS-MPLs (0.1 and 1 μm) for 24 h, PS-MPLs (0.1 μm) were observed in the cytoplasm of the cells. . A significant increase in the ALT, AST, and MDA levels and a decrease in GSH and liver pathological scores at 0.1 and 1 mg/L of PS-MPs exposure indicates that MPLs were toxic to hepatocytes. PS-MPLs also damaged the nucleus and induced micronucleus formation in hepatocytes. A low dose of 1 mg/L of PS-MPLs was not cytotoxic to HL7702 cells; hence these cells have been experimented with for long-term intervention. With increasing time, the expression of γH2ax, ATM, and 53BP1 in these cells was significantly increased. Immunofluorescent staining showed that the γH2ax foci elevated dramatically in micro-PS (MPLs) treated cells, although most cells' morphology presented no noticeable differences when compared with normal cells. In addition, the number of micro-nuclei in the cytoplasm that had slipped out of the nucleus and were tagged with H2ax, increased. A decrease in cell metabolism and mitochondrial regulators (PPARα and PGC-1α), mitochondrial fusion proteins (MFN-1), and complexes I and III (ND1 and UQCRC2, respectively) and ATP levels in the liver demonstrates mitochondrial damage and dysfunction in liver cells by PS-MPLs. The significant enrichment of mtDNA, including mt-Co1, mt-Nd6, and mt-Cyb, was observed by qPCR analysis of cytosolic fractions (without mitochondria) of HL7720 cells in the cytoplasm, which showed that PS-MPLs cause mtDNA leakage into the cytoplasm. Because of this leakage, the expression of DNA sensor cGAS and the downstream proteins STING and the *p*-NFκB response was also increased, demonstrating that PS-MPLs cause the cGAS/STING pathway activation. The NF-κB target gene pro-inflammatory cytokines (IL-1β, IL-6, and TNFα), liver fibrosis marker (α-SMA), and levels of fibronectin were also increased. In addition, STING was transferred to membraniform structures around the nucleus.^236^ This crucial study confirmed that the activation of the cGAS/STING pathway initiated the downstream cascade reaction, and NF-κB was translocated into the nucleus and upregulated pro-inflammatory cytokines expression, thus facilitating liver fibrosis eventually.^105^

### Cell death: Apoptotic pathways

Apoptosis is a programmed cell death^237^ that helps the body to eliminate damaged cells beyond repair. M-NPLs have been demonstrated to induce apoptosis through p53, PI3K/AKT, and Bcl-2/Bax apoptotic pathways.^108^^,^^238^ Umamaheswari et al.^238^ treated *D. rerio* (Zebrafish) with 10μg L^−1^ and 100μg L^−1^ of PS-MPLs having a size of 0.10–0.12 μm for 35 days and reported that gene expression for TNF-α, p53, casp3b, gadd45ba, and ptgs2a was significantly upregulated. Histological examination of gill tissue also indicated cytoplasmic degradation, aneurysm, necrosis, lamellar fusion, and epithelial lifting in PS-MPs treated groups. These results suggest that PS-MPs-induced ROS production enhances peroxidation resulting in the production of HNE (4-Hydroxynonenal) and MDA (malondialdehyde), promoting DNA adduct formation and hence cell death. ROS also upregulates p53 genes causing the activation of casp3b, which causes the transcription of gadd45ba, causing DNA damage and apoptosis. This leads to tissue injury, resulting in increased expression of ptgs2a and TNF-α (a pro-inflammatory cytokine) and release of LDH enzymes. On the other hand, ROS generation suppresses the expression of cytoprotective genes such as cat, sod1, and gpx1a while increasing the expression of the detoxifying gene gstp1, regulating antioxidant translation. To sum it up, PS-MPLs induced activation of the p53 pathway, dysregulation of enzymes (LDH, AChE, CAT, and SOD) and cytoprotective genes, and inflammatory responses are responsible for apoptosis in Zebrafish.^238^

**Figure 8:**
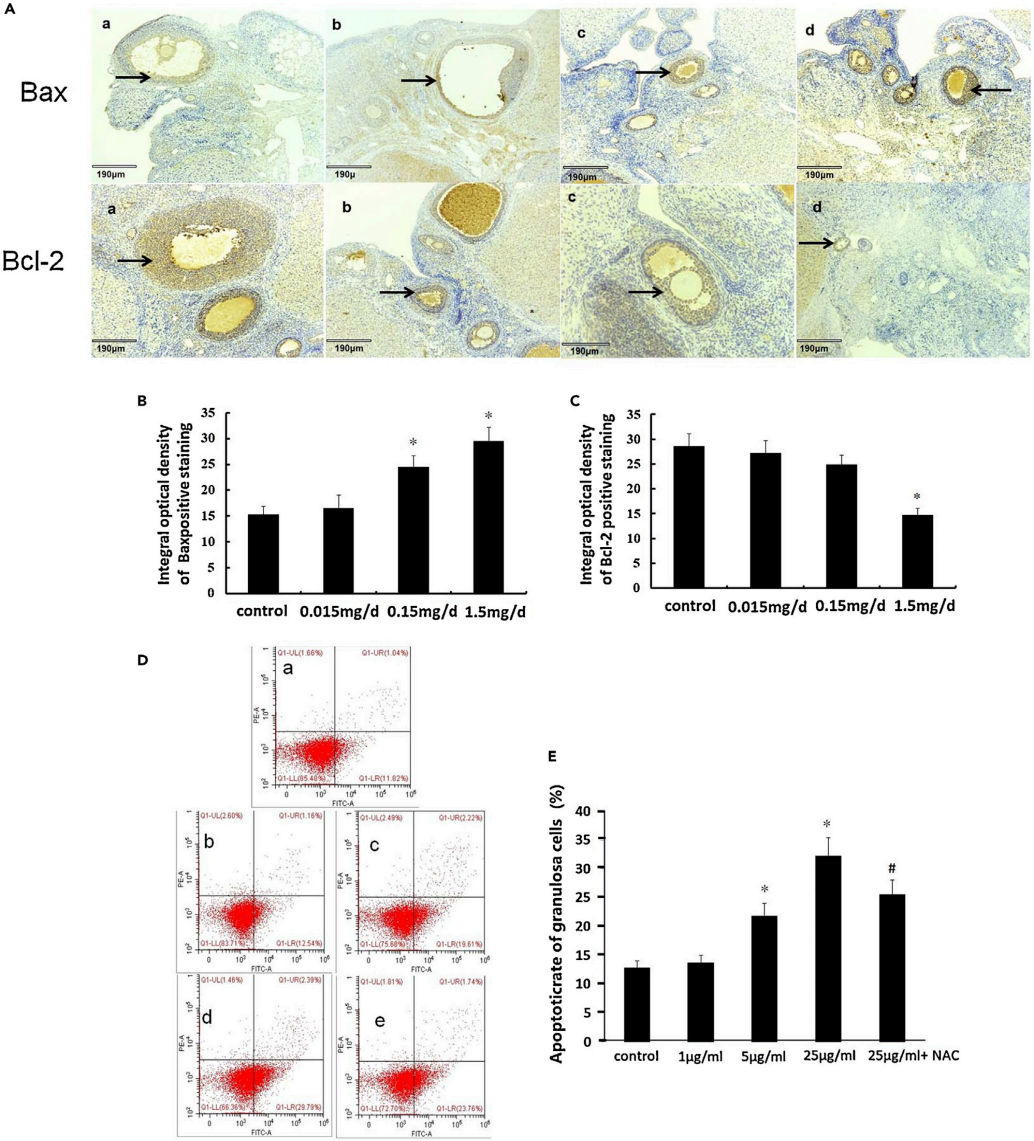
Effects of PS-MPLs on Bax and Bcl-2 expression in the ovary and apoptosis in GCs in rats (A–E) Immunohistochemical analysis of Bcl-2 and Bax expression (A). The various groups in Fig A are the control group (a), PS-MPLs 0.015 (b), PS-MPLs 0.15 (c), and PS-MPLs 1.5 mg/d (d). Bcl-2 and Bax expressions’ IOD values were determined by the Image-pro plus 6.0 (B and C). Apoptotic rate (D) and percent apoptotic rate (E) in GCs after PS-MPLs treatment were determined by flow cytometer using PI- and FITC-labeled Annexin V double staining. Adopted with permission from Elsevier.^102^

Qin et al.^108^ reported that wastewater disinfection involving chlorine treatment makes the surface of water containing PS-MPs rough and induces apoptosis through PI3K/AKT and Bcl-2/Bax pathways in Human gastric epithelial (GES-1) cells. They used PS-MPs (smooth surface, 213 nm size), L-Cl_2_-PS-MPs (PS-MPLs treated with 10 mg/L for 21 days, rough surface with cracks and pits, 213 nm size), and H-Cl_2_-PS-MPs (PS-MPs treated with 100 mg/L for 21 days, rough surface with crack and pits, 213 nm size). Only about 4.4 and 9.6% of the cells in the control and pristine PS-MPLs treated cells were in apoptosis after 48 h of exposure, respectively. On the other hand, on exposure to L-Cl2-PS-MPs and H-Cl2-MPs, roughly 12.6% and 24.2% of populations went into apoptosis.^108^ These findings suggest that water chlorination causes excessive apoptosis in GES-1 cells, disrupting the gastric barrier, which is linked to digestive failure and even the development of gastric disorders,^108^^,^^239^ as shown in Figure 8. To explore the molecular mechanism, the effect of these modified plastic particles on the PI3K/AKT and BCL-2/Bax pathway was assessed. A decrease in the expression of PI3K (promoter of cell proliferation and defense against apoptosis), *p*-AKT (Thr 308), and Bcl-2 and an increase in the expression of Bax, Bax/Bcl-2 ratio, cleaved caspase-9, and cleaved caspase-3 after exposure to chlorinated PS-MPLs was observed. These results suggest that chlorinated PS-MPLs execute apoptosis through PI3K/AKT and Bcl-2/Bax pathways.

NPLs can also inhibit Caco-2 cell proliferation, which is suspected to be associated with increased ROS, mitochondrial damage, and apoptosis because of NPLs-induced lysosomal rupture and instability.^101^ Enhanced apoptosis and reduced granulosa cell number have been seen in rats treated with PS-MPLs. Bax protein, an inducer of apoptosis expression, showed increased levels, whereas Bcl-2 protein, which inhibits apoptosis by increasing time-to-death and intrinsic cell-to-cell differences in the mitochondrial mechanism of cell death, was reduced, indicating that PS-MPLs cause apoptosis in rats,^102^ as displayed in Figure 8.

#### The role of microplastics and nanoplastics in cell membrane disruption

M-NPLs have been reported to interact with the cell membrane, causing cellular toxicity. Lactate Dehydrogenase (LDH), MDA release, and lipid peroxidation (LPO) can be used as biomarkers to observe cell membrane disruption. Lactate dehydrogenase (LDH) is a cytoplasmic enzyme that turns sugar into energy and is found in every cell of the body. As soon as cells are damaged, LDH is released into the fluid, blood, or media and can be measured through LDH assay.^240^ Xu et al.^101^ observed the effects of NPLs on the cell membrane integrity using LDH assay for Caco-2 cells seeded in a 96-well plate at a density of 1 × 10^4^ cells/well. These cells were treated with NPLs for 24 or 48 h and then analyzed by spectrophotometer. No significant increase in LDH release was observed, indicating that NPLs did not damage the cell membrane integrity.^101^ Nevertheless, other studies have reported cell membrane disruption by M-NPLs exposure. For instance, both 100 nm and 500 nm PS-NPLs have been seen to damage the cell membrane (increase LDH) of HUVECs at different concentrations (5- 25 μg/L) and exposure times (10 min- 3 h).^144^

MDA is a biomarker of oxidative stress and an end product of LPO of polyunsaturated fatty acids. Thiobarbituric acid reactive substances (TBARS) assay is commonly used to measure MDA.^241^ Lipid peroxidation is a chain of oxidative lipid degradation where free radicals acquire electrons from lipids in the cell membrane, resulting in membrane damage.^242^ PS-MPLs (10μg L^−1^ and 100μg L^−1^) have been shown to increase LPO levels in a time and dose-dependent manner compared to the control group.^102^ The level of MDA was also increased in the same pattern. Granulosa cells (GC) treated with 0.15 and 1.5 mg/d PS-MPLs also increase the level of MDA, indicating these MPLs ' toxic effects on the granulosa cells.^102^

#### The role of microplastic and nanoplastics in inducing autophagy

Autophagy is a highly conserved cell degradation process that occurs naturally using a lysosome-dependent and controlled mechanism to eliminate unwanted or defective components.^243^ M-NPLs can induce cell death (toxicity) utilizing the autophagic pathway. The enhanced fluorescence caused by NPLs localization both inside and outside the lysosome and the rise in the number of lysosomes following NPLs treatments indicates higher absorption of NPLs by cells and the participation of lysosomes in NPLs-induced autophagy.^101^ Bafilomycin A1, a lysosome maturation inhibitor and lysosome-endosome fusion inhibitor, decreases NPLs fluorescence in Caco-2 cells, indicating that inhibiting autophagic pathways (lysosome maturation and fusion) reduces macropinocytosis and Clathrin-mediated endocytosis mediated absorption of NPLs.^101^

**Figure 9:**
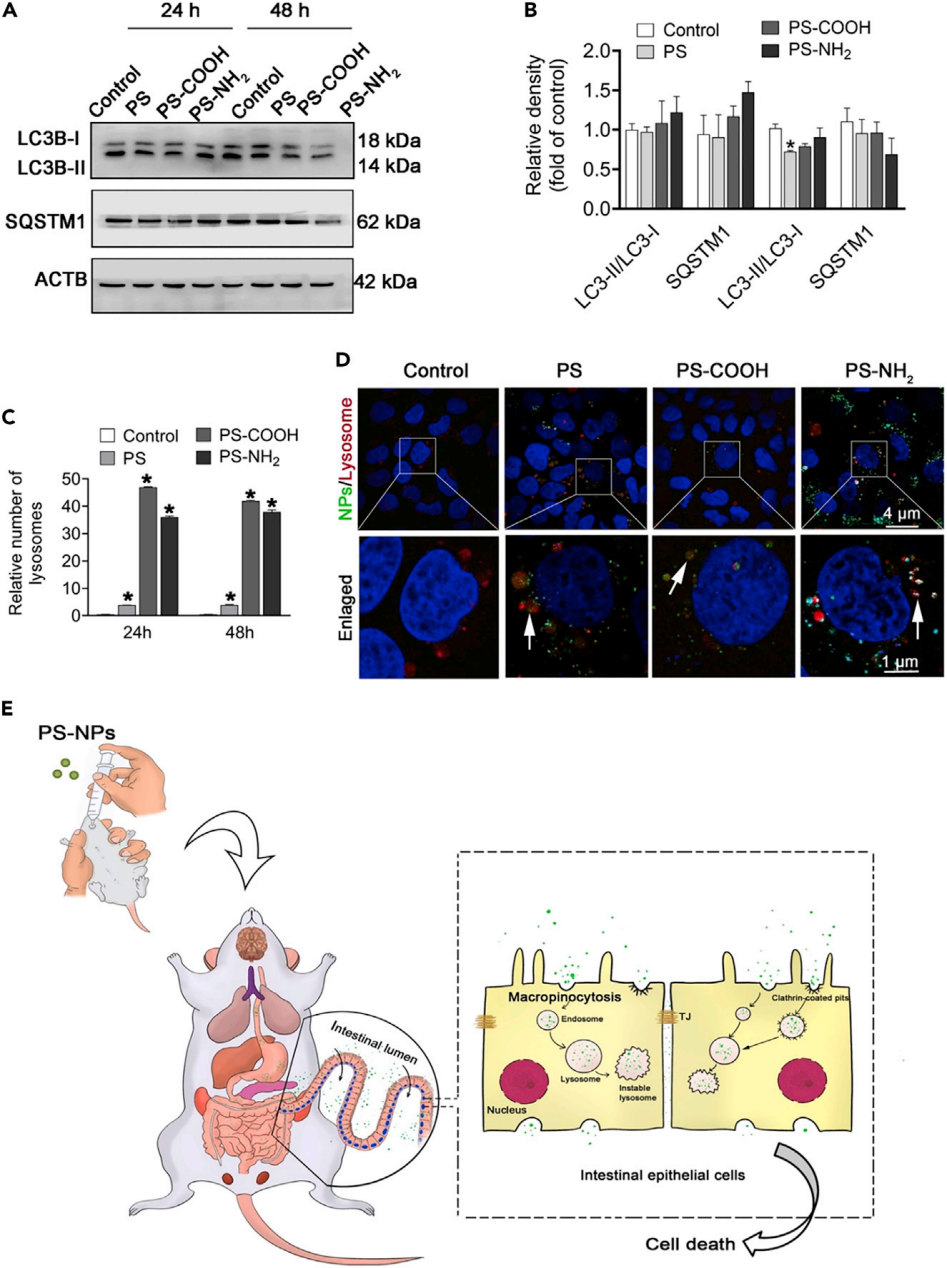
Caco-2 cells treated with NPLs showed increased lysosome levels (A–E) Analysis of LC3-II and SQSTM1 proteins (A, B). Flowcytometry detection of lysosome tagged with LysoTracker red (C). Confocal microscopy analysis of lysosome (D). Sketch the molecular mechanism for NPL internalization in Caco-2 cells (E). Adopted with permission from Elsevier.^101^

NPLs were also found to cause autophagic cell death in Caco-2 cells.^244^^,^^245^ Following NPLs treatment, autophagic cell death markers such as the Bax/Bcl-2 ratio, LC3-II (autophagosome marker), and SQSTM1 protein levels increased in Caco-2 cells,^101^ as shown in Figure 9. The Bax/Bcl-2 ratio is a credible parameter as it acts as a rheostat, i.e., determining cell susceptibility to apoptosis.^246^ Chen et al.^107^ investigated the nephrotoxic effects of round-shaped PS-MPLs (3.54 0.39 μm) at environmental concentrations in HEK293 cells and reported that PS-MPLs adhered to the cell membrane and were absorbed entirely by HEK293 cells. PS-MPLs induced cytotoxicity through oxidative stress by suppressing the antioxidant heme oxygenase-1. The proportions of autophagic cells were determined by DAP Green staining. The HEK293 cells were cultured with DAP Green dye for 30 min at 37 °C after 24 h of exposure to PS-MPLs (300 ng/mL). In autophagic cells, DAP Green dye exhibits green fluorescence (emission at 530 nm). PS-MPLs were found to induce apoptosis and autophagy simultaneously by depolarizing the mitochondrial membrane potential and forming autophagosomes. The inflammatory factors were only triggered by a non-cytotoxic concentration of PS-MPLs (3 ng/mL) (33 cytokines). In contrast, the cytotoxic concentration of PS-MPLs (300 ng/mL) promoted autophagy, which may further lower the NLR family pyrin domain containing 3 (NLRP3) expression, hence decreasing inflammation (35 cytokines) in HEK293 cells.^107^

##### M-NPLs as vectors of environmental pollutants and emerging risks

M-NPLs act as vectors of pollutants such as heavy metals^247^ and persistent organic pollutants (POPs), including phthalate (PAEs), polybrominated diphenyl Ethers (PBDEs), polychlorinated biphenyls (PCBs), polycyclic aromatic hydrocarbons (PAHs) and perfluoroalkyl substances (PFAS) in terrestrial and aquatic ecosystems.^248^^,^^249^^,^^250^ MPLs adsorb, transport, and release pollutants into the local tissue, as observed in aquatic species such as rainbow fish, marine amphipods, larval zebrafish, and mussels.^251^^,^^252^^,^^253^ M-NPLs present in the ocean for a long time can get enriched with environmental organic pollutants through a series of complex interactions. M-NPLs can then carry these pollutants into living organisms. M-NPLs can also enhance the toxicity of organic pollutants when they coexist. Brennecke et al.^247^ observed the adsorption of heavy metals (copper and Zinc) onto pristine PS-MPLs released from antifouling paint in water, hence supporting the role of M-NPLs as heavy metal carriers. It has also been reported that MPLs can load high amount of various model heavy metals in a sequence of Pb^2+^>Cu^2+^>Cd^2+^.^254^ PCBs and PBDEs have been found in fish fed with marine plastic but not in those fed with virgin plastic, suggesting that plastic debris is a vector for pollutants to wildlife.^255^ Deng et al.^250^ reported in his pioneer study that M-NPLs can release phthalate esters (PAEs) and cause exacerbated effects in the mouse gut. Results of this study also showed differential regulation of 703 genes after 30 days of exposure to DEHP-contaminated MPLs. Furthermore, an increase in intestinal permeability, inflammation, induced immune responses, oxidative stress, and disturbed metabolism^250^ was also observed. In addition, gut microbiota analysis revealed that combined MPLs and DEHP co-exposure resulted in a relative abundance of energy metabolism and immune function-related gut bacteria.^250^ PFAS are a large class of chemicals used in various everyday products. The carbon-fluorine bonds of PFAS make them difficult to degrade in the environment. A recent study examined the adsorption capacity of 18 PFASs by three different types of microplastics: high-density polyethylene (HDPE), polystyrene (PS) and polystyrene carboxylic acid (PS-COOH).^256^ The results revealed that all three MPLs (HDPE, PS and PS-COOH) can adsorb and stabilize residues of PFASs present in the surrounding water.^256^ Overall, PS and PS-COOH have more affinity for PFASs than HDPE. In addition, perfluoro sulfonates and sulfonamides are also more readily absorbed onto MPLs.^256^ It was suggested that hydrophobic interactions and salting out are the main influencing factors in this adsorption process. Hydrophobic interactions increase the adsorption affinity of MPLs for longer-chain compounds and the solution pH can alter the surface charge of MPLs.^256^ These studies provide useful information about the health risks of MPLs as carriers of various pollutants.

## Insights into the toxicity of microplastics and nanoplastics and their relevance to COVID-19

Several investigations have found a substantial link between COVID-19 and M-NPLs.^257^ Plastic is the primary component of all types of surgical face masks and thus can be a significant source of M-NPLs release.^91^^,^^257^ SARS-CoV-2 RNA was found in the air around a large medical center in Brazil, and it was linked to the amount of MPLs fibers in the air and high temperatures and humidity. Hence, MPLs could act as a carrier of the SARS-CoV-2 virus, increasing virus survivability in the atmosphere and thereby promoting virus entry into the human body.^258^ The Elovich equation has described the release kinetics of microplastics,^259^ stating that MPLs with a diameter of less than 500 μm demonstrate a significant release quantity and rate.^259^ The production of surgical masks has also been reported to rise in tandem with the rise in positive COVID-19 cases, rendering it a potential source of M-NPLs litter and contaminant in soil, air, and water. Three layers of surgical face masks were disassembled and found to contain plastic polymers.^257^ Also, wearing a mask elevates the risk of M-NPLs inhalation, and reusing a mask increases this risk. Regardless of whether a mask is used, the risk of inhaling spherical-type M-NPLs released from the facemask remained significant. However, N95 performed well in lowering spherical and fiber-like microplastic inhalation compared to other masks and no masks.^260^ Shen et al.^261^ found that discarded surgical masks can leak microplastics into the environment. In addition, it was discovered that mask aging released billions of microplastics into the atmosphere. Adding detergent and alcohol solution to the mask enhanced its release of microplastics.^261^ The increased usage of wet wipes was also a substantial cause of microplastic pollution during the global COVID-19 pandemic.^262^ Excessive use of facemasks manufactured of polypropylene materials that incorporate meltblown face mask filters (MB filters) and the environmental burden of littered used facemasks are two severe issues currently.^262^ The nanofibers created from microfibers and fragments of MB filters of facemasks contribute to the dust overload in the body when these M-NPLs are inhaled while wearing a mask. This may have dangerous consequences.^262^ This subject needs to be examined in detail, and more research should be carried out.

Therefore, an individual’s best judgment regarding mask use is vital as face masks are a prominent source of M-NPLs release and inhalation. For instance, a person with a beard should consider replacing masks regularly because the beard may contribute to mask’s physical abrasion of microplastics. Overall, although face masks may lead to microplastic inhalation, the use of face masks should not be opposed as it is critical during a pandemic, however, best practice is recommended while using face masks.

## Future prospects and conclusions

M-NPLs are becoming a severe environmental issue, and a recent boost in M-NPLs research is evident. However, we are only at the very beginning of our understanding and exploration of the problem; there are still many questions in the field:

• Nomenclature and abbreviation issue of M-NPLs
• The impact of M-NPLs on the gut microbiota and, consequently, human physiology
• How M-NPLs biotransform *in vivo*
• Limited research on M-NPL mixtures in terms of different types, sizes, charges, and surface groups.
• The role of M-NPL *in vivo* is poorly understood, lacking any clinical and epidemiological studies

The first issue to settle is the naming and abbreviation issue of Microplastics and Nanoplastics. Different names for the same M-NPLs can cause various errors and confusion in the scientific community. The debate surrounding the problematic alternative use of the word nanoplastics and nanoparticles must be discussed. Because nanoparticles are defined as particles with a diameter of 1–100 nm,^263^ all plastic particles in the 1–100 nm range are classified as nanoplastics. For instance, Yong et al.^264^ used the abbreviation “NPs” for nanoplastics. On the contrary, later in the study, when they illustrated the mechanism behind MPLs/NPLs toxicity, they used literature on nanoparticles to demonstrate nanoplastics. Two of the references in acute or chronic toxicity in mammalian cells are from nanoparticle studies instead of microplastics or nanoplastics. Therefore, we suggest consistent abbreviated forms, such as NPLs for nanoplastics, PS-NPLs for polystyrene nanoplastics, MPLs for microplastics, PS-MPLs for polystyrene microplastics, and M-NPLs for microplastics and nanoplastics to prevent misunderstanding. Furthermore, sufficient clarification should be provided when referring to nanoparticles or nanoplastics.

It is well known that the gut microbiota has been utilized as a toxicological target for various environmental toxins that can modify the host’s physiological processes by affecting the gut microbiota’s structure.^228^^,^^265^ Stressors-induced changes in gut microbiota can also lead to various diseases, including and not limited to metabolic disorders.^266^ Studies have reported the microbial degradation of MPLs by algae, fungi, and bacteria.^267^ Although enzymes derived from microorganisms have been examined for MPLs degradation,^267^ the breakdown of various MPLs by these enzymes is unknown. Because interacting with environmental pollutants and gastrointestinal tract (GIT) microbes is a two-way process, understanding and exploring this will reveal answers to different idiopathic causes of disorders such as schizophrenia. Furthermore, exploring this bidirectional interaction will also provide insights into the impact of MPLs on human health, GIT M-NPLs tolerance, and alternatives to reduce M-NPL’s burden on GIT. Unfortunately, no *in vivo* studies have been conducted on the role of GIT inhibitory microorganisms in M-NPLs degradation and the harmful consequences of these M-NPLs on gut microbiota. Research on biotransformation and detoxifying the toxic effects of M-NPLs in the body is yet to be initiated. Biotransformation of M-NPLs by the body, particularly the liver, kidney, and lungs, which are known to transform lipophilic chemicals into hydrophobic substances, has still not been explored. The scientific community should also focus on whether M-NPLs, like most xenobiotics, require the phase I stage of biotransformation, and if so, how cytochromes P450 enzymes play a role, and whether in the phase II stage glutathione conjugation, glucuronide conjugation, sulfate conjugation, acetylation, and methylation contribute to biotransformation of M-NPLs.

Furthermore, little is known about the *in vivo* effects of M-NPLs. Most of the literature published is based on *in vitro* studies using cell lines, and a small number of *in vivo* studies exist using model animals, particularly mice. Moreover, the M-NPLs used in these studies were not comparable to the concentration of M-NPLs detected in human blood (1.6 μg/mL).^62^*In vivo* studies are required to investigate the effects of different types, concentrations, and surface characteristics of M-NPLs in various model animals, notably higher primates. It is also worth noting that M-NPLs in the environment are often heterogeneous with respect to type, size, concentration, charge, and surface groups. Hence, additional research into the possible impacts of the mixture of different M-NPLs could help researchers better understand their potential exposure hazards. This would also aid in determining the severity of M-NPLs toxicity and pollution in the environment and develop an acceptable biological range to assess M-NPLs toxicity. Furthermore, more studies are needed to determine the release of M-NPLs both *in vitro* and *in vivo*, as only one study has shown that M-NPLs are released via lysosomal exocytosis. Further research in this area will help determine the extent of toxicity *in vivo* and understand the fate of M-NPLs in cells, organ systems, and, ultimately, the human body. Similarly, the trans-generational consequences of M-NPLs on organisms remain vastly unknown. Only a single study has used hIPSCs to investigate the impact of M-NPLs on embryogenesis. The data in this field will reveal the component of genetic heredity linked to M-NPLs toxicity.

M-NPLs have been recovered from various human samples, and different *in vitro* cell culture models have provided mechanisms of action for M-NPLs toxicity. However, clinical and epidemiological studies are needed to explore M-NPLs toxicity. These studies would hold tremendous implications for developing risk assessment guidelines for human exposure to M-NPLs.

In summary, this article provides the most recent review of the toxic effects of M-NPLs at the organ, cellular, and organelle levels through ingestion, inhalation, and dermal exposure. M-NPLs have been detected in human blood, lungs, sputum, saliva, hair, face skin, hand skin, stool, and placenta samples. Following exposure, M-NPLs get distributed through blood, transcellular, and paracellular transport. When M-NPLs approach cells, they interact with cell membranes via various bonds (e.g., hydrogen, halogen) or hydrophobic, van der Waals, and electrostatic forces. M-NPLs can be toxic to nearly every organ system (for example, the reproductive, respiratory, nervous, endocrine, and cardiovascular systems). After cellular internalization via endocytosis (macropinocytosis, clathrin, and caveolae-mediated endocytosis), M-NPLs have been shown to interact with and accumulate in various organelles (for example, the nucleus, mitochondria, lysosomes, and ER). This article also reviewed the mechanism of M-NPLs toxicity. M-NPLs toxicity is associated with reactive oxygen species (ROS) generation, oxidative stress, nuclear factor-erythroid factor 2-related factor 2 (Nrf2), and the Wnt/β-Catenin signaling pathways. In addition, inflammation has also been identified as one of the most notorious manifestations of M-NPLs exposure, possibly due toM-NPLs-induced pro-inflammatory cytokines involving the activation of Nuclear Factor Kappa B (NF-κB) and cGas/STING pathways. M-NPLs can also induce autophagy, cell membrane disruption, and cell apoptosis. Finally, a significant role of M-NPLs in exacerbating the COVID-19 pandemic has been discussed, as plastic particles that loaded the virus into the air increased the half-life of the virus and facilitated the transmission of the virus to humans through the Trojan horse effect. Increased transmission and, consequently, more cases of COVID-19 will lead to increased production and use of surgical masks, an acknowledged source of M-NPLs.
